# Implications of food ultra-processing on cardiovascular risk considering plant origin foods: an analysis of the UK Biobank cohort

**DOI:** 10.1016/j.lanepe.2024.100948

**Published:** 2024-06-10

**Authors:** Fernanda Rauber, Maria Laura da Costa Louzada, Kiara Chang, Inge Huybrechts, Marc J. Gunter, Carlos Augusto Monteiro, Eszter P. Vamos, Renata Bertazzi Levy

**Affiliations:** a Department of Preventive Medicine, School of Medicine, University of São Paulo, São Paulo, 01246 903, Brazil; b Center for Epidemiological Research in Nutrition and Health, University of São Paulo, São Paulo, 01246-904, Brazil; c Department of Nutrition, School of Public Health, University of São Paulo, São Paulo, 01246-904, Brazil; d Public Health Policy Evaluation Unit, School of Public Health, Imperial College London, London, W6 8RP, United Kingdom; e Nutrition and Metabolism Branch, International Agency for Research on Cancer, Lyon, 69372, France; f Department of Epidemiology and Biostatistics, School of Public Health, Imperial College London, London, W2 1PG, United Kingdom

**Keywords:** Ultra-processed food, Plant-based food, Cardiovascular disease, Mortality

## Abstract

**Background:**

Comprehensive research evidence is lacking on the role of ultra-processed foods (UPF) in the relationship between the consumption of plant-sourced foods and their impact on cardiovascular disease (CVD) outcomes. This study aims to assess CVD risk associated with the dietary contribution of food groups that consider both plant or animal origin and food processing categories, within a large cohort of British adults.

**Methods:**

Data from the UK Biobank participants (40–69 y) who completed at least two 24-h dietary recalls between 2009 and 2012 (n = 126,842; median follow-up: 9 y), with subsequent data linkage to hospital and mortality records, were used. Food groups were classified as either plant-sourced or non-plant/animal-sourced foods. These groups were further divided into non-UPF and UPF, and expressed as a percentage of total energy intake.

**Findings:**

Every 10 percentage points increase in plant-sourced non-UPF consumption was associated with a 7% lower risk of CVD (95% CI 0.91–0.95) and a 13% lower risk of CVD mortality (0.80–0.94). Conversely, plant-sourced UPF consumption was associated with a 5% increased risk (1.03–1.07) and a 12% higher mortality (1.05–1.20). The contribution of all UPF was linked to higher CVD risk and mortality, and no evidence for an association between contribution of all plant-sourced foods and CVD incidence and mortality was observed.

**Interpretation:**

The dietary contribution of plant-sourced non-UPF inversely linked to CVD risk, while plant-sourced UPF contribution showed a positive association. Recognizing the role of food processing is crucial for favourable CVD outcomes, even in plant-sourced diets.

**Funding:**

10.13039/100011713World Cancer Research Fund.

**Research in context**

**Evidence before this study**

Plant-sourced dietary patterns, which are characterized by low consumption or complete omission of eggs, dairy, fish, and meat, have been linked to a reduced risk of various chronic diseases, including cardiovascular disease (CVD). In May 2024, a PubMed search was conducted using the following search terms: “ultra-processed foods” (and its variations: “ultraprocessed foods,” “ultra-processed food,” “ultraprocessed food”), “cardiovascular diseases” (including “cardiovascular mortality” and “cardiovascular health”), and “plant-based diet” (or “plant-based”). Our search did not yield any prospective studies that specifically examined the role of ultra-processed foods in the relationship between the consumption of plant-sourced foods and their impact on CVD outcomes.

**Added value of this study**

This is the first large-scale cohort study to simultaneously consider the degrees of industrial food processing and food sources (plant versus animal) on CVD risk. This study shows that the dietary contribution of plant-sourced non-ultra-processed foods is associated with lower risks of CVD, while the contribution of plant-sourced ultra-processed foods is associated with higher risks. It is important to note that the dietary contribution of all plant-sourced foods is not associated with CVD risk and the dietary contribution of all ultra-processed foods is associated with higher CVD risk. In addition, we found that replacing intake of plant-sourced UPF with plant-sourced non-UPF was associated with a 7% and 15% lower risk of CVD incidence and CVD-cause mortality, respectively.

**Implications of all the available evidence**

Dietary guidelines promoting diets based on plant-sourced foods should emphasize not only the reduction of meat, red meat, or animal-sourced foods but also the need to avoid all ultra-processed foods.

## Introduction

Cardiovascular disease (CVD) remains the leading cause of premature mortality across the globe, contributing to 18.6 million deaths in 2019.^1^ In the United Kingdom, around 7.6 million people are living with CVD, which accounts for a quarter of all deaths.^2^ CVD currently costs the UK economy (including premature death, disability and informal costs) an estimated £19 billion each year.^2^ Among all modifiable risk factors for CVD, the promotion of healthy dietary patterns is probably one of the most cost-effective strategies to prevent CVD.^3^

Plant-sourced dietary patterns, as characterized by low consumption or complete omission of eggs, dairy products, fish, and meat, have been associated with a reduced risk of several chronic diseases, as well as a substantial reduction in impacts on the environment.^4^ There has been an increase in the consumption of plant-sourced alternative foods in recent years, with a two-fold increase in the proportion of people reporting consuming these products in the UK.^5^ In 2019, the UK Climate Change Committee recommended a 20% reduction in high-carbon meat and dairy products by 2030, with an increased consumption of plant-sourced products.^6^ These recommendations are in line with national and international guidelines for a healthy diet that guide the reduction of meat consumption, especially red meat.^7^ However, plant-sourced dietary patterns are heterogeneous and may differ widely in their dietary composition, type, and quality,^8^ and evidence has shown the potential protective effect of plant-sourced diets on CVD may vary accordingly.^9^, ^10^, ^11^

Modern plant-sourced diets may incorporate a range of ultra-processed foods (UPF), such as sugar-sweetened beverages, snacks, confectionery, but also the ‘plant-sourced’ sausages, nuggets, and burgers that are produced with ingredients originating from plants and marketed as meat and dairy substitutes. UPF, the fourth group of the Nova classification system, are industrial formulations made by deconstructing whole foods into chemical constituents, altering and then recombining them with additives into products that are alternatives to the other three Nova groups and freshly prepared dishes and meals based on them.^12^ While these three Nova groups (unprocessed/minimally processed foods, culinary ingredients, and processed foods) include foods commonly found in traditional diets worldwide, some of which are associated with health and longevity, UPF is identified as a distinct group that poses health risks.^12^ A recent comprehensive systematic umbrella review, which included evidence from 45 pooled analyses encompassing almost 10 million participants, found that a greater exposure to ultra-processed food was associated with a higher risk of 32 health parameters, including cardiometabolic disorders, common mental health disorders, and mortality.^13^ Although the exact mechanisms through which UPF may harm health are not fully understood, their unbalanced nutritional composition (commonly high in fat, sugar, and salt and poor in fibre and micronutrients) the novel physical structures and chemical compositions of UPF, including those that claim to be plant-sourced, are possible mechanisms.^14^

To date, comprehensive research evidence is lacking on the role of UPF in the association between plant-sourced food consumption and CVD outcomes. Therefore, the primary objective of our study was to assess the potential risk of CVD associated with the dietary contribution of plant-sourced diets using the UK Biobank, while differentiating them based on the contribution of UPF. Specially, we examined the associations of consuming plant-sourced non-UPF and plant-sourced UPF on CVD risk and CVD-related mortality. Additionally, we conducted a similar analysis focusing on the dietary contribution of non-red meat, which involved omitting only red meat from this group. We also differentiated these non-red meat items based on the contribution of UPF. This approach was prompted by previous research indicating that reducing the consumption of red meat, rather than all types of meat, may be associated with a reduced risk of CVD.^15^

## Methods

### Study design and participants

UK Biobank is a large prospective cohort study that recruited over 500,000 participants aged 40–69 years at baseline (between 2007 and 2010) across England, Scotland, and Wales with data linkage to hospital and mortality records. At baseline assessment, participants completed a self-administered touch-screen questionnaire on their socio-demographic, lifestyle (e.g., history of smoking) and health-related information (e.g., family history of CVD). Participants’ physical measurements (e.g., height, weight) were collected by trained staff following standardised procedures. Further details of all measurements can be found in the UK Biobank online protocol (http://www.ukbiobank.ac.uk).

The UK Biobank received ethical approval from the North West Multi-centre Research Ethics Committee (21/NW/0157) and data access was granted by the UK Biobank's Access Subcommittee. At recruitment, all participants gave informed consent to participate and be followed-up through data-linkage.

### Procedures

Dietary intakes were assessed using a validated web-based, self-administered questionnaire designed to record the consumption of over 200 common food and beverage items in the previous 24 h. This 24-h recall was introduced towards the end of the recruitment period (2009–2010). All participants with a known email address were invited to complete the questionnaire online on four separate occasions between 2011 and 2012. For these analyses, food items consumed by participants were characterized based on the proportion of total energy intake from plant-sourced versus non-plant/animal-sourced foods. Subsequently, these two groups were further divided into the proportion of energy intake from non-UPF versus UPF.

We considered plant-sourced foods as all foods exclusively or primarily of plant origin (e.g., fruits, vegetables, grains, breads). Animal-sourced foods included all meats (i.e., fish, poultry, red meats, etc.), dairy products, and eggs. Supplementary Table S1 details examples of foods for each category.

For the food processing category, we used the Nova classification system, which considers the extent and purpose of the food manufacturing process.^12^ The derivation of individual dietary consumption by the degree of industrial food processing has been documented in detail elsewhere.^16^ In brief, we applied the Nova classification to 24-h recall data assigning each food and beverage item to one of the four main food groups: 1) unprocessed or minimally processed foods, e.g., fresh, dry or frozen fruits or vegetables; grains, flours and pasta; pasteurized or power plain milk, plain yogurt, fresh or frozen meat; 2) processed culinary ingredients, e.g., table sugar, oils, butter, and salt; 3) processed foods, e.g., vegetables in brine, cheese, simple breads, fruits in syrup, canned fish; and 4) UPF, e.g., soft drinks, sweet or savoury packaged snacks, confectionery; packaged breads and buns; reconstituted meat products and pre-prepared frozen or shelf-stable dishes. For this study, we estimated the proportion of total dietary energy from non-UPF (the first three groups of Nova classification) versus UPF.

The dietary contributions of plant-sourced non-UPF, plant-sourced UPF, all plant-sourced foods and all UPF were categorised into quartiles (% of total energy) and also assessed as continuous (per 10% increase in total energy contribution) variables.

### Outcomes assessment

Incident cardiovascular disease was defined as the first hospital admission or death (primary cause) from cardiovascular diseases using International Classification of Diseases (ICD)-10 codes which included coronary heart disease (I20.0, I21, I22 and I25) and cerebrovascular disease (I60–I64 and G45). Fatal CVD events were identified from mortality files using the same ICD-10 codes. The hospital registry-based follow-up ended on 30th September 2021, in England; 24th September 2021, in Scotland; and 31st May 2016, in Wales. Death registry included all deaths that occurred before 31st December 2020 in England, Wales, and Scotland.

### Covariates

Baseline study covariates included: age, sex (male, female), ethnicity (white, non-white), region (London, South East, South West, East Midlands, West Midlands, Yorkshire & the Humber, North East, North West, Wales, Scotland), Index of Multiple Deprivation (IMD; quintile), body mass index (BMI) (continuous), physical activity (low, moderate, high, missing), smoking status (never smoked, ex-smoker, current smoker) and family history of cardiovascular disease (no, mother or father, mother and father). IMD is a composite measure of deprivation for each small area of the UK based on participants’ postcode, and we derived IMD quintiles based on deprivation scores.

Participants with missing covariates data were excluded, except for physical activity and IMD variables. Since 16,614 (14%) and 3009 (2.5%) participants had missing data on physical activity and IMD variables, respectively, we included a missing class into the models for these variables to preserve sample size and reduce the risk of selection bias.

The selection of confounding variables for inclusion in the analysis model was based on a thorough review of the literature and theoretical considerations, focusing on variables consistently associated with the exposure and outcome of interest.

### Statistical analyses

**Fig. 1:**
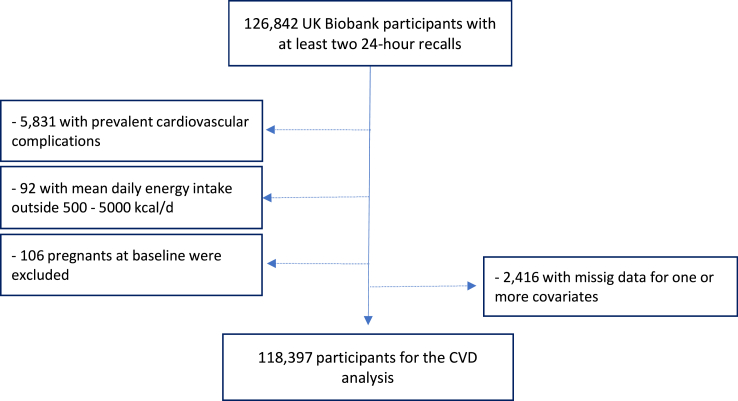
**Flow diagram for cardiovascular complications analysis**.

For this study, we included participants with at least two 24-h dietary recalls collected (n = 126,842). We excluded participants with cardiovascular disease at baseline (n = 5831), with a total daily energy intake outside of the predefined limits (<500 kcal and >5000 kcal) (n = 92), women who were pregnant at baseline or became pregnant during the follow-up period (n = 106), and participants with missing data for one or more covariates (n = 2416). Data from 118,397 participants were included in the analyses (Fig. 1), and the mean of all available days of food recall for each person were used to estimate the dietary contribution of plant-sourced non-UPF and plant-sourced UPF.

We examined the characteristics of the study population at baseline and by quartiles of the proportion of dietary energy from plant-sourced non-UPF and plant-sourced UPF. Group differences by quartiles of food contribution were assessed using analysis of variance or χ2 tests as appropriate.

We visually inspected the graphical representations of the survival functions by quartiles of plant-sourced food contribution and categories of other covariates using Kaplan–Meier plots. We assessed the equality of survival distributions between subgroups using log rank tests. We used Cox proportional hazards regression models with age as the underlying time metric to estimate the hazard ratios and their corresponding 95% confidence intervals for the incidence of each outcome for each quartile of food contribution considering the lowest quartile as the reference (or as a continuous variable as described above). We developed separate models to assess the impact of plant-sourced non-UPF and plant-sourced UPF. These models were adjusted for sex, ethnicity, family history of cardiovascular disease, BMI, physical activity, smoking status, IMD, and region. The proportional hazards assumption of Cox regression model was verified by testing the Schoenfeld residuals against survival time. These analyses revealed a violation of the proportionality assumption for sex, family history of cardiovascular diseases and smoking status (fatal and non-fatal event models) and for sex and ethnicity (fatal event models), therefore, a stratification of those variables was applied to the models. In all models, time at entry was age at recruitment and participants were followed up until the date of cardiovascular disease diagnosis, end of study period, loss to follow-up, or death, whichever occurred first. The interactions between the diet variables (plant-sourced non-UPF, plant-sourced UPF, animal-sourced non-UPF, and animal-sourced UPF) were tested by adding a multiplicative term in the Cox regression models but this was not found statistically significant (data not shown). Linear trend was assessed across the quartiles. We verified the assumption of linearity between the food groups and risk of cardiovascular diseases using restricted cubic spline functions.

Since the dietary variables studied (plant-sourced non-UPF, plant-sourced UPF, animal-sourced non-UPF, and animal-sourced UPF) represent compositional data in terms of percentage intake, a substitution analysis was performed. To assess the effect of replacing 10% of each of the three food groups (presumably less healthy) with 10% of plant-sourced non-UPF on cardiovascular disease risk, we used Cox proportional hazards regression models. In the model for each outcome, three food groups were included, with the fourth group serving as the reference. The hazard ratio estimate represented the substitution of every 10% of each of the three food groups with an equal amount of plant-sourced non-UPF, while keeping the other groups constant. The models were constructed exclusively for the outcomes that demonstrated associations with the exposures investigated in the primary analyses, and the same covariates were adjusted for.

For further analysis, food items consumed by participants were also characterized based on the proportion of total energy intake from non-red meat (all plant-sourced foods plus fish, poultry, dairy products, and eggs) versus red meat. These two groups were then further divided into the proportion of energy intake from non-UPF versus UPF. Subsequently, all analyses were repeated to assess the dietary contribution of non-red meat non-UPF and non-red meat UPF as exposure.

The following sensitivity analyses were also performed: additionally adjusting for (i) animal-sourced UPF, (ii) red meat UPF, (iii) alcohol intake (g/day), (iv) free sugars (% of total energy), saturated fat (% of total energy), sodium density (mg/1000 kcal), and fibre density (g/1000 kcal), (v) pre-existing type 2 diabetes (yes or no) and high blood pressure (yes or no); (vi) considering food groups as a proportion of daily grams intake (% of total grams) and additionally adjusting for total daily energy intake (kcal/day); (vii) excluding participants with a follow-up time <2 years.

All statistical analyses were conducted using Stata version 14.0 and a p-value of <0.05 was considered statistically significant.

### Role of the funding source

The funders of the study had no role in study design, data collection, data analysis, data interpretation, or writing of the report.

## Results

Among the 118,397 participants (57.1% females), the mean age at baseline was 55.9 ± 7.8 years. Table 1 shows the main baseline characteristics of participants according to quarters of the contribution of plant-sourced non-UPF in diet. Compared with participants in the lower quartile, those in the upper quartile tended to be older, were more likely to be female, non-white and ex- or current smokers, had a lower mean BMI and higher physical activity levels, and were more likely to live in the least deprived area. By contrast, the characteristics of participants according to plant-sourced UPF contribution showed an opposite profile (Supplementary Table S2). Compared with participants in the lower quartile, those in the upper quartile tended to be younger, were more likely to be male and non-white, were less likely to have a family history of CVD, had a higher mean BMI, have lower physical activity levels, have never smoked, and were more likely to live in the most deprived area.

**Table 1:** Characteristics of the study population according to quartiles of the dietary contribution of plant-sourced non-ultra-processed foods, UK Biobank cohort (n = 118,397).

	All participants	Quartile of the dietary contribution of plant-sourced non-ultra-processed foods (mean % of total energy)				p value^a^
		1 (16.2%)	2 (25.9%)	3 (33.6%)	4 (46.0%)	
*mean (SD) or % (n)*						
**Baseline age, years**	55.9 (7.8)	55.4 (8)	56.0 (7.9)	56.2 (7.7)	56.1 (7.6)	*<0.001*
**Female sex**	57.1 (67,551)	56.3 (16,671)	57.6 (17,043)	57.3 (16,947)	57.1 (16,890)	*0.021*
**Ethnicity white**	96.9 (114,725)	97.7 (28,179)	97.2 (28,779)	96.9 (28,684)	95.8 (28,360)	*<0.001*
**Family history of CVD**						
No	45.1 (53,447)	45.7 (13,519)	45.0 (13,315)	44.7 (13,233)	45.2 (13,380)	*0.205*
Yes, mother or father	42.1 (49,844)	41.9 (12,404)	42.1 (12,447)	42.5 (12,576)	42.0 (12,417)	
Yes, mother and father	12.8 (15,106)	12.4 (3677)	13.0 (3837)	12.8 (3790)	12.9 (3802)	
**Baseline BMI status, kg/m**^**2**^	26.6 (4.6)	27.5 (5)	26.7 (4.6)	26.3 (4.3)	25.9 (4.1)	*<0.001*
**Pre-existing type 2 diabetes**	3.4 (3972)	4.6 (1360)	3.4 (1016)	2.9 (855)	2.5 (741)	*<0.001*
**Pre-existing high blood pressure**	22.3 (26,428)	24.2 (7164)	22.5 (6648)	21.4 (6340)	21.2 (6276)	*<0.001*
**Physical activity**						
Low	15.7 (18,580)	19.3 (5726)	16.2 (4801)	14.5 (4281)	12.7 (3772)	*<0.001*
Moderate	37.1 (43,914)	35.3 (10,461)	38.0 (11,244)	37.5 (11,111)	37.5 (11,098)	
High	33.2 (39,289)	28.7 (8489)	31.6 (9353)	34.5 (10,204)	38.0 (11,243)	
Missing	14.0 (16,614)	16.6 (4924)	14.2 (4201)	13.5 (4003)	11.8 (3486)	
**Smoking status**						
Never smoked	58.0 (68,717)	61.7 (18,258)	60 (17,770)	57.7 (17,085)	52.7 (15,604)	*<0.001*
Ex-smoker	35.2 (41,616)	30.7 (9096)	33.6 (9949)	36.2 (10,716)	40.1 (11,855)	
Current smoker	6.8 (8064)	7.6 (2246)	6.4 (1880)	6.1 (1798)	7.2 (2140)	
**Index of multiple deprivation**						
1st quintile (least deprived)	19.8 (23,387)	17.6 (5204)	20.9 (6181)	20.7 (6138)	19.8 (5864)	*<0.001*
2nd quintile	19.5 (23,099)	17.8 (5261)	20.0 (5905)	20.5 (6076)	19.8 (5857)	
3rd quintile	19.6 (23,159)	19.4 (5753)	19.8 (5856)	19.8 (5872)	19.2 (5678)	
4th quintile	19.4 (22,975)	20.1 (5956)	19.3 (5701)	18.9 (5597)	19.3 (5721)	
5th quintile (most deprived)	19.2 (22,768)	22.7 (6705)	17.7 (5235)	17.5 (5169)	19.1 (5659)	
Missing	2.5 (3009)	2.4 (721)	2.4 (721)	2.5 (747)	2.8 (820)	
**Geographical region**						
London	20.6 (24,369)	14.8 (4390)	18.2 (5375)	21.5 (6364)	27.8 (8240)	*<0.001*
South East	9.4 (11,091)	8.7 (2581)	9.5 (2812)	9.8 (2892)	9.5 (2806)	
South West	10.4 (12,322)	9.8 (2904)	10.7 (3154)	10.7 (3168)	10.5 (3096)	
East Midlands	6.2 (7319)	7.0 (2070)	6.5 (1918)	6.2 (1833)	5.1 (1498)	
West Midlands	7.9 (9348)	9.2 (2707)	8.3 (2445)	7.6 (2255)	6.6 (1941)	
Yorkshire & the Humber	15.4 (18,181)	16.9 (5013)	15.8 (4675)	15.0 (4431)	13.7 (4062)	
North East	9.4 (11,174)	11.3 (3346)	9.9 (2938)	8.9 (2637)	7.6 (2253)	
North West	12.3 (14,532)	13.8 (4072)	12.7 (3748)	11.8 (3481)	10.9 (3231)	
Wales	3.1 (3701)	3.4 (1009)	3.2 (953)	3.0 (900)	2.8 (839)	
Scotland	5.4 (6360)	5.1 (1508)	5.3 (1581)	5.5 (1638)	5.5 (1633)	
**Nutrients**						
Total energy (kcal)	2034 (538)	2162 (596)	2077.5 (531)	2006 (500)	1890 (479)	*<0.001*
Free sugars (% of energy)	13.5 (6.5)	15.7 (7.4)	14.1 (6.3)	12.8 (5.8)	11.5 (5.6)	*<0.001*
Saturated fats (% of energy)	10.9 (3.0)	12.5 (3.0)	11.4 (2.8)	10.6 (2.7)	9.1 (2.6)	*<0.001*
Fibre (g/1000 kcal)	12.6 (4.4)	11.6 (4.4)	12.2 (4.0)	12.7 (4.1)	13.8 (4.8)	*<0.001*
Sodium (mg/1000 kcal)	935 (218)	1020 (224)	956 (199.1)	914 (199)	850 (212)	*<0.001*

BMI, Body Mass Index.

a Analysis of variance or χ2 test where appropriate.

The mean contribution of all plant-sourced foods to the overall diet (in kcal/day) was 69.9%, being 30.5% non-UPF and 39.4% UPF. Regarding the rest of the diet, 21.4% came from animal-sourced non-UPF and 8.8% from animal-sourced UPF (Table 2).

**Table 2:** Dietary contribution (% of total energy intake) of foods grouped according to both plant or animal origin and food processing categories. UK Biobank cohort (n = 118,397).

Plant-sourced foods	%	SD	Animal-sourced foods	%	SD
**Non-ultra-processed**	**30.5**	**11.8**	**Non-ultra-processed**	**21.4**	**8.6**
Fruit	8.9	5.7	Red meat^b^	4.6	4.8
Beer and Wine	5.8	6.9	Milk	4.3	3.7
Cereals	3.7	4.5	Fish	3.1	4.8
Vegetables	2.5	1.9	Cheese	3.1	3.2
Pasta	2.2	3.8	Poultry	2.5	3.3
Roots and tubers	1.7	2.1	Animal fats	2.1	3.3
Processed bread	1.7	3.3	Eggs	1.7	2.6
Nuts and seeds	1.2	2.5			
Table sugar	0.8	2.1			
Vegetables/fruit preserved	0.7	0.9			
Legumes	0.6	1.5			
Others^a^	0.5	1.2			
**Ultra-processed**	**39.4**	**13.2**	**Ultra-processed**	**8.8**	**8.0**
Industrialised packaged breads	9.9	5.8	Milk-based drinks	4.2	6.8
Pastries, buns, and cakes	6.9	6.8	Sausage and other reconstituted red meat products^b^	1.5	3.0
Biscuits	3.9	4.6	Nuggets and other reconstituted meat products	1.3	3.0
Margarine and other spreads	3.3	3.0	Milk based desserts	1.0	1.7
Industrial chips (French fries)	2.8	3.9	Mayonnaise and spreadable cheese	0.7	1.6
Confectionery	2.7	3.7			
Breakfast cereals	2.7	3.2			
Soft drinks, fruit drinks, and fruit juices	2.0	3.3			
Packaged salty snacks	1.7	2.6			
Industrial pizza	1.3	4.8			
Packaged pre-prepared meals	0.9	1.6			
Distilled alcoholic drink	0.8	2.3			
Sauces, dressing and gravies	0.3	0.5			
Meat alternatives	0.2	1.0			
**Total**	**69.9**	**10.3**	**Total**	**30.1**	**10.3**

a Coffee and tea, fungi, homemade soup, plant oil.

b Considered as red meat in the further analyses using non-red meat versus red meat, according to dietary contribution of UPF.

### UPF, plant-sourced foods and cardiovascular disease incidence and mortality

The associations between the dietary contribution of foods groups that consider both the plant or animal origin of foods and food processing categories (% of total energy), and fatal and non-fatal cardiovascular events are shown in Table 3. The linearity assumption between intake of each diet category and each outcome was assessed using restricted cubic spline (Supplementary Table S3). No statistically significant violation from the linearity assumption was observed except for all plant-sourced foods and mortality for all CVD (p = 0.04) and for coronary heart disease (p = 0.03).

**Table 3:** Association between the dietary contribution of foods groups that take into account both the plant or animal origin of foods and food processing categories, and fatal and non-fatal cardiovascular events from in the UK biobank cohort (n = 118,397).

Food groups	Dietary contribution (% of total energy)					
	Q1	Q2	Q3	Q4	p for trend	Continuous (10% increase in the contribution)
	HR (95% CI)					HR (95% CI)
**All cardiovascular diseases**						
*n for cases/non-cases = 7806/110,591*						
Plant-sourced non-UPF	1	0.89 (0.84–0.95)	0.85 (0.80–0.91)	0.80 (0.75–0.86)	*<0.001*	0.93 (0.91–0.95)
Plant-sourced UPF	1	1.05 (0.98–1.12)	1.15 (1.07–1.22)	1.16 (1.09–1.24)	*<0.001*	1.05 (1.03–1.07)
All plant-sourced foods	1	0.99 (0.93–1.05)	0.95 (0.89–1.01)	0.97 (0.91–1.04)	*0.229*	0.99 (0.97–1.02)
All UPF	1	1.09 (1.02–1.17)	1.17 (1.10–1.25)	1.23 (1.15–1.31)	*<0.001*	1.06 (1.04–1.08)
**Coronary heart disease**						
*n for cases/non-cases = 6006/112,391*						
Plant-sourced non-UPF	1	0.89 (0.83–0.95)	0.84 (0.78–0.90)	0.77 (0.71–0.83)	*<0.001*	0.92 (0.90–0.94)
Plant-sourced UPF	1	1.09 (1.01–1.17)	1.20 (1.12–1.29)	1.21 (1.13–1.31)	*<0.001*	1.06 (1.04–1.09)
All plant-sourced foods	1	1.02 (0.95–1.09)	0.96 (0.89–1.03)	0.98 (0.91–1.05)	*0.243*	0.99 (0.97–1.02)
All UPF	1	1.18 (1.09–1.27)	1.24 (1.15–1.34)	1.31 (1.21–1.41)	*<0.001*	1.07 (1.05–1.09)
**Cerebrovascular disease**						
*n for cases/non-cases = 2112/116,285*						
Plant-sourced non-UPF	1	0.91 (0.81–1.03)	0.93 (0.83–1.05)	0.93 (0.82–1.05)	*0.310*	0.99 (0.95–1.03)
Plant-sourced UPF	1	0.94 (0.84–1.06)	0.96 (0.85–1.09)	1.03 (0.91–1.16)	*0.602*	1.01 (0.98–1.05)
All plant-sourced foods	1	0.92 (0.82–1.04)	0.95 (0.84–1.07)	0.97 (0.86–1.10)	*0.727*	1.00 (0.96–1.05)
All UPF	1	0.89 (0.79–1.01)	0.98 (0.87–1.10)	1.00 (0.88–1.13)	*0.697*	1.01 (0.98–1.04)
**All cardiovascular diseases mortality**						
*n for cases/non-cases = 529/117,868*						
Plant-sourced non-UPF	1	0.90 (0.72–1.13)	0.78 (0.62–0.99)	0.61 (0.47–0.79)	*<0.001*	0.87 (0.80–0.94)
Plant-sourced UPF	1	1.00 (0.77–1.31)	1.39 (1.08–1.78)	1.49 (1.16–1.92)	*<0.001*	1.12 (1.05–1.20)
All plant-sourced foods	1	1.05 (0.83–1.33)	0.89 (0.70–1.13)	0.89 (0.70–1.14)	*0.195*	1.00 (0.92–1.09)^a^
All UPF	1	1.30 (1.00–1.68)	1.35 (1.04–1.74)	1.42 (1.10–1.84)	*0.010*	1.09 (1.02–1.16)
**Coronary heart disease mortality**						
*n for cases/non-cases = 348/118,049*						
Plant-sourced non-UPF	1	0.84 (0.64–1.10)	0.71 (0.53–0.94)	0.48 (0.34–0.66)	*<0.001*	0.80 (0.73–0.88)
Plant-sourced UPF	1	1.32 (0.93–1.87)	1.75 (1.26–2.44)	1.90 (1.37–2.65)	*<0.001*	1.18 (1.09–1.28)
All plant-sourced foods	1	1.09 (0.81–1.45)	0.86 (0.64–1.17)	0.86 (0.63–1.16)	*0.151*	0.99 (0.89–1.10)^a^
All UPF	1	1.49 (1.07–2.07)	1.64 (1.18–2.27)	1.65 (1.19–2.28)	*0.004*	1.13 (1.05–1.23)
**Cerebrovascular disease mortality**						
*n for cases/non-cases = 181/118,216*						
Plant-sourced non-UPF	1	1.06 (0.71–1.59)	0.97 (0.64–1.47)	0.95 (0.62–1.46)	*0.742*	1.01 (0.89–1.15)
Plant-sourced UPF	1	0.67 (0.43–1.04)	1.00 (0.67–1.49)	1.05 (0.70–1.57)	*0.472*	1.00 (0.89–1.13)
All plant-sourced foods	1	0.99 (0.66–1.47)	0.93 (0.62–1.41)	0.97 (0.64–1.48)	*0.827*	1.02 (0.88–1.18)
All UPF	1	1.06 (0.70–1.60)	0.97 (0.63–1.48)	1.13 (0.75–1.72)	*0.670*	1.01 (0.90–1.12)

Q, Quartile; UPF, Ultra-processed foods.

Mean follow-up times were 9.1 for overall cardiovascular disease (1,076,104 person-years), 9.2 coronary heart disease (1,083,490 person-years), and 9.3 for cerebrovascular diseases (1,101,715 person-years). Mean follow-up times were 9.2 for mortality for cardiovascular disease (1,091,678 person-years), coronary heart disease (1,091,678 person-years), and cerebrovascular diseases (1,091,678 person-years).

Cut-off for quarters of food contribution ranged from 30.3% of total energy intake (1st quartile) to 65.9% (4th quartile) for UPF; from 56.3% to 82.1% for plant-based foods; from 16.3% to 46.0% for plant-based foods non-UPF; and from 22.9% to 56.4% for plant-based foods UPF, respectively.

Cox proportional hazards models with age as the underlying timescale. Adjusted by sex, ethnic (white, non-white), family history of CVD (no, mother or father, mother and father), BMI (continuous), physical activity (low, moderate, high, missing), smoking status (never, previous, current), index of multiple deprivation (quintile), and region (London, South East, South West, East Midlands, West Midlands, Yorkshire & the Humber, North East, North West, Wales, Scotland). Analysis for risk of CVD were stratified by sex, family history of CVD and smoking status. Analysis for the CVD death were stratified by sex and ethnic.

a Non-linear association in restricted cubic spline regression (p = 0.04 and p = 0.03, respectively).

A total of 7806 incident CVD cases occurred during 1,076,104 person-years of follow-up (mean, 9.1 years), including 6006 coronary heart events and 2112 cerebrovascular events. After adjustment for potential confounders, a 10% increase in the contribution of plant-sourced non-UPF in diet was associated with a 7% reduced risk of incident CVD (adjusted HR 0.93; 95% CI 0.91–0.95) and a 8% reduced risk of incident coronary heart disease (adjusted HR 0.92; 95% CI 0.90–0.94); while plant-sourced UPF contribution was associated with an increased risk of both outcomes (adjusted HR 1.05; 95% CI 1.03–1.07 for all CVD; and adjusted HR 1.06; 95% CI 1.04–1.09 for coronary heart disease). A higher dietary contribution of UPF overall was associated with an increased risk of all CVD (adjusted HR for a 10% increase in the contribution: 1.06; 95% CI 1.04–1.08) and coronary heart disease (adjusted HR 1.07; 95% CI 1.05–1.09); while there was no evidence of an association of the all plant-sourced food contribution with any CVD outcomes.

A total of 529 CVD deaths occurred during 1,091,678 person-years of follow-up (median, 9.2 years), including 348 coronary heart disease deaths and 181 cerebrovascular deaths. After adjustment for potential confounders, a 10% increase in the dietary contribution of plant-sourced non-UPF was associated with a 13% lower mortality of all CVD (adjusted HR: 0.87; 95% CI 0.80–0.94) and a 20% lower mortality of coronary heart disease (adjusted HR 0.80; 95% CI 0.73–0.88); while plant-sourced UPF contribution was associated with a higher risk of mortality for all CVD (adjusted HR for 10% increase 1.12; 95% CI 1.05–1.20) and coronary heart disease (adjusted HR 1.18; 95% CI 1.09–1.28). The dietary contribution of all UPF was associated with a higher mortality of all CVD (adjusted HR for a 10% increase in the contribution: 1.09; 95% CI 1.02–1.16) and coronary heart disease (adjusted HR 1.13; 95% CI 1.05–1.23); while there was no evidence of association between all plant-sourced food contribution and cardiovascular deaths.

The analyses using quartiles of the dietary contribution showed consistent trends with the results of the analysis using continuous variables (per 10% increase in the contribution). In summary, participants in the highest quartile of plant-sourced non-UPF contribution presented a lower incidence and mortality of CVD and coronary heart disease compared to those in the lowest quartile of contribution. Conversely, participants in the highest quartile of plant-sourced UPF contribution presented a higher incidence and mortality for both outcomes.

There was no evidence of an association observed between any of the food groups and cerebrovascular incidence or mortality.

**Fig. 2:**
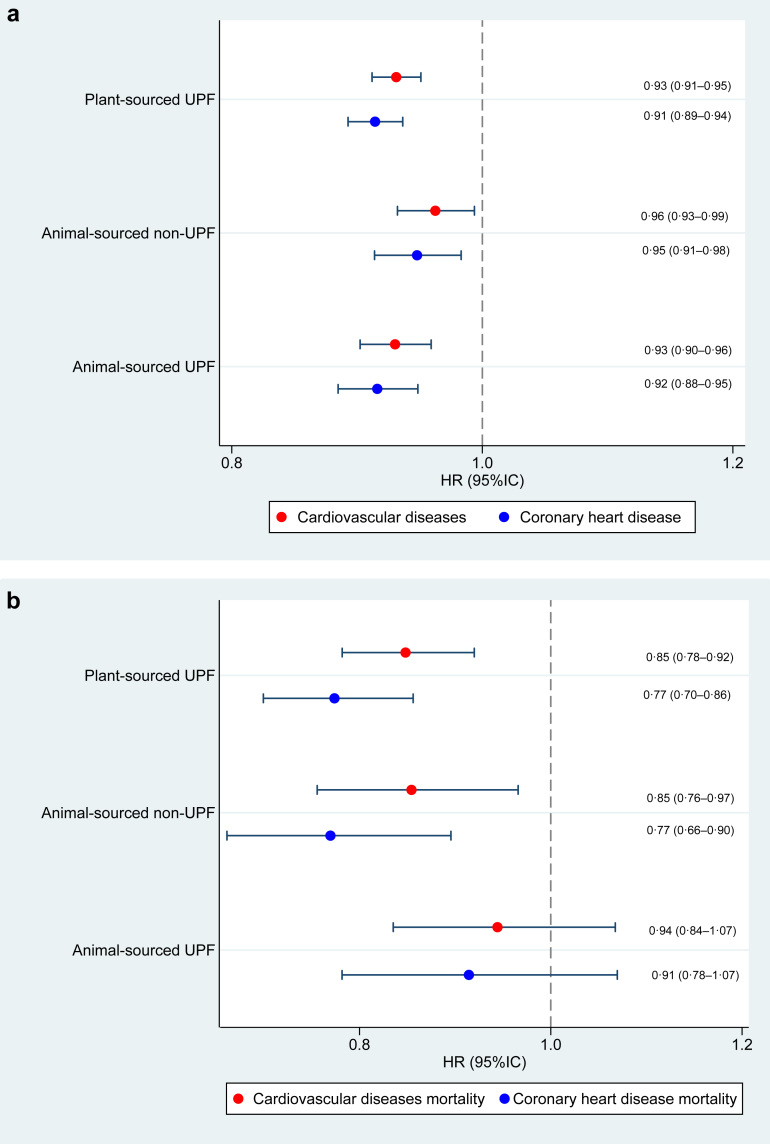
**Effect of replacing 10% of each of the 3 food groups (plant-sourced UPF, animal-sourced non-UPF, and animal-sourced UPF) with plant-sourced non-UPF**. Note: Food substitutions among UK Biobank participants (n = 118,397). Fully adjusted hazard ratios (HR) and 95% confidence intervals (CI) were calculated using Cox proportional hazards regression to assess the substitutions of contributions from food groups that take into account both plant or animal origin of foods as well as food processing categories, and their associations with cardiovascular incidence (Fig. 2a) and cardiovascular mortality (Fig. 2b). All results are from continuous linear models. Plant-sourced ultra-processed by animal-sourced non-ultra-processed or animal-sourced ultra-processed. Animal-sourced non-ultra-processed by animal-sourced ultra-processed. UPF, ultra-processed foods.

In our substitution analysis (Fig. 2), replacing 10% of any of the three food groups (plant-sourced UPF, animal-sourced non-UPF, or animal-sourced UPF) with an equal amount of dietary energy from plant-sourced non-UPF was associated with a reduced risk of incident CVD and coronary heart disease. The substitution models yielded similar results in the mortality analysis, except for the replacement of dietary energy from animal-sourced UPF with plant-sourced non-UPF, which did not reach significance, although the HR indicated some level of protection.

### Additional analyses and further adjustments

The analysis considering the dietary contribution of non-red meat corroborated the principal findings (Supplementary Table S4 and Supplementary Figure S1).

Sensitivity analyses including additional adjustments for animal-sourced UPF and red meat UPF, alcohol intake, nutrient intake and pre-existing type 2 diabetes and hypertension; using daily grams intake from food groups; and excluding participants with <2 years follow-up were all consistent with the initial findings (Supplementary Tables S5–S11).

## Discussion

Our analyses of the large UK Biobank cohort study revealed important associations between the consumption plant-sourced foods, considering the degree of food processing, and the risk of CVD. We observed that higher dietary contribution of plant-sourced non-UPF were associated with a lower risk of fatal and nonfatal cardiovascular events, while contribution of plant-sourced UPF was associated with a higher risk of cardiovascular events. This pattern of associations was also evident regarding CVD-specific mortality. In addition, we found that replacing intake of plant-sourced UPF with plant-sourced non-UPF was associated with a 7% and 15% lower risk of CVD incidence and CVD-cause mortality, respectively. Finally, our study reveals that the influence of the dietary contribution of non-red meat on CVD risk also depends on food processing. These findings advance current knowledge by highlighting that a higher intake of plant-sourced foods may only bring about better cardiovascular health outcomes when largely based on minimally processed foods while a higher intake of plant-sourced UPF may have detrimental effects on health.

Previous studies have found a beneficial effect in adopting a healthful plant-sourced diet and reduced CVD risk.^9^, ^10^, ^11^ However, none of these studies have clearly assessed whether the degree of industrial food processing affects this association. This is particularly important when considering a possible rising trend in new plant-sourced ultra-processed products. A study conducted with participants from the NutriNet-Santé cohort revealed that vegetarians and vegans consumed more UPF than meat eaters, primarily through the consumption of industrial plant-sourced meat and dairy substitutes.^8^ Emerging evidence has shown many harmful health effects associated with UPF consumption,^13^ this study provides evidence for the first time that the impact of plant-sourced UPF on CVD should not be overlooked.

Despite being plant-sourced, UPF-rich diets may still pose health risks due to negative effects caused by their composition and processing methods. High content of unhealthy fats, sodium, and added sugars in UPF contribute to dyslipidemia, atherosclerosis, hypertension, insulin resistance, obesity, and metabolic disorders,^13^ all CVD risk factors. Notably, results of our sensitivity analyses that further adjusted for these nutrients remained significant, suggesting other non-nutritional factors may have contributed to the associations, consistent with previous studies.^17^ Certain food additives found in UPF, such as monosodium glutamate and artificial sweeteners, as well as contaminants formed during industrial processing, such as acrolein, have been associated with an increased risk of CVD, possibly through oxidative stress, inflammation, endothelial dysfunction, metabolic dysregulation, insulin resistance, and alterations in gut microbiota composition.^18^, ^19^, ^20^ Absence of an intact food matrix in plant-sourced UPF may lead to lower levels of bioactive compounds (e.g., polyphenol and phytosterols),^21^ that are associated with CVD risk reduction.^22^ Additionally, plant constituents such as fibre may beneficially affect the composition and function of the large intestinal microbiome, and bacterial metabolites that may be associated with CVD.^23^^,^^24^

In a study conducted by Orlich and collegues,^25^ higher consumption of UPF was associated with an approximately 14% increase in all-cause mortality rate, even in a health-conscious population with a substantial number of vegetarians (over half of the participants). While no evidence for a significant association was found for total animal-sourced food intake, moderate consumption of red meat showed an 8% increased risk. In our study, which focused on a population with a higher proportion of meat-eaters and animal food consumers, the relationship between the dietary contribution of non-red meat (including plant-sourced foods, fish, poultry, dairy products, and eggs) and CVD risk was dependent on its UPF status.

These findings are in line with previous meta-analyses, which consistently demonstrate a significant positive association between the consumption of processed meat and various CVD and mortality outcomes.^26^^,^^27^ However, the relationship between unprocessed red meat intake and health outcomes varies across studies. In a recent investigation utilizing data from the UK Biobank, higher consumption of unprocessed red meat was found to be associated with an increased risk of CVD mortality.^28^ Notably, our study differs in certain aspects, such as the utilization of multiple 24-h recalls, which provide more accurate identification of food processing levels and estimation of daily amounts of red meat and other foods, in contrast to the food frequency questionnaire that assesses weekly intake of red meat. Moreover, our study is distinguished by the rigorous application of the Nova food classification criteria,^29^ which may elucidate potential discrepancies observed in comparison to previous studies. For instance, while salted, cured, or smoked meats are typically categorized as processed meats, they may not always fall under the classification of UPF according to Nova.

Finally, the lack of statistically significant results for cerebrovascular disease incidence and especially for cerebrovascular disease mortality may be partly due to the relatively small number of events for these outcomes. Future studies to further evaluate these associations are warranted.

Some notable strengths of the study include the large sample size and prospective design, enhancing the robustness of the findings. Additionally, the minimum of two validated 24-h recall questionnaire ensures reliable and accurate assessment of dietary patterns. Furthermore, the Nova food classification system is a widely recognized approach that utilizes standardized and objective criteria to classify foods based on their level of processing.^29^ Finally, this is the first large-scale cohort study to simultaneously consider the degrees of food processing and food sources (plant versus animal and red versus non-red meat products).

Potential limitations should be considered. Firstly, 24-h recall are susceptible to recall bias, misreporting, and the accuracy of food composition databases. However, the online administration likely reduced reporting bias due to social desirability, and extreme values of total calorie intake were excluded from the analysis. Secondly, the prospective design reduces potential risk for reverse causality and our sensitivity analyses excluding participants followed up for less than 2 years confirmed the robustness of the associations. Thirdly, despite adjusting for important confounders, residual confounding cannot be completely ruled out. Finally, despite of the low response rate (approximately 9.2 million invitations were sent out to recruit the targeted sample size of 0.5 million), the characteristics of the cohort and estimated effect sizes resemble closely to those of the general population.^30^ However, this may limit the generalizability of summary statistics and absolute risk estimates.

### Conclusion

The findings of this large UK cohort study indicate that higher dietary contribution of plant-sourced non-UPF may be associated with a lower risk of CVD. These results support the notion to improve CVD health outcomes with a shift towards plant-sourced food choices that consider the degree of food processing. Our findings also demonstrated that the relationship between the dietary contribution of non-red meat (all foods, except red meat) and CVD risk depended on whether it underwent ultra-processing or not. Future research and dietary guidelines promoting a plant-sourced diet should emphasize not only the reduction of meat, red meat, or animal-sourced foods but also the need to avoid all UPF.

## Contributors

FR, MLCL, CAM and RBL conceptualised the study and FR, MLCL, KC, CAM, EPV and RBL contributed to the study design. FR compiled the data and performed statistical analyses. All authors contributed to the finalisation of statistical models and interpretation of findings. FR wrote the first draft of the manuscript, and MLCL, KC, IH, MJG, CAM, EPV and RBL critically reviewed and edited the manuscript. All authors had full access to all the data in the study, approved the final manuscript, and accept responsibility for the decision to submit for publication. The corresponding authors attest that all listed authors meet authorship criteria and that no others meeting the criteria have been omitted.

## Data sharing statement

UK Biobank data are available through application to the database.

## Declaration of interests

We have no competing interests to declare.
