# Supplementary material for: Implications of food ultra-processing on cardiovascular risk considering plant origin foods: an analysis of the UK Biobank cohort
Source: Lancet Reg Health Eur. 2024 Jun 10;43:100948. doi: 10.1016/j.lanepe.2024.100948 (PMC11360147; doi:10.1016/j.lanepe.2024.100948)
Supplement: Supplemental material [file mmc1.docx]

**Supplemental material**

**Implications of food ultra-processing on cardiovascular risk considering plant origin foods: an analysis of the UK Biobank cohort**

Fernanda Rauber, Maria Laura da Costa Louzada, Kiara Chang, Inge Huybrechts, Marc J Gunter, Carlos Augusto Monteiro, Eszter P Vamos, Renata Bertazzi Levy

Table S1: Examples of food items considered in each food group.

Table S2: Characteristics of the study population according to quartiles of dietary contribution of plant-sourced ultra-processed foods.

Table S3: Test for linearity assumption between intake of each food groups and each outcome using restricted cubic spline regression.

Table S4 and Figure S1: Further analysis the association between the dietary contribution of food groups that consider both the red meat or non-red meat origin of foods and food processing categories, and fatal and non-fatal cardiovascular events.

Table S5: Sensitivity analysis for the association between the dietary contribution of foods groups that take into account both the plant or animal origin of foods and food processing categories, and fatal and non-fatal cardiovascular events, additionally adjustment for animal-sourced ultra-processed foods and for red meat ultra-processed foods.

Table S6: Sensitivity analysis for the association between the dietary contribution of foods groups that take into account both the plant or animal origin of foods and food processing categories, and fatal and non-fatal cardiovascular events, additionally adjustment for alcohol, nutrients and baseline status for type 2 diabetes and high blood pressure.

Table S7: Sensitivity analysis for the association between the dietary contribution of food groups that take into account both the red meat or non-red meat origin of foods and food processing categories and fatal and non-fatal cardiovascular events, additionally adjustment for alcohol, nutrients and baseline status for type II diabetes and high blood pressure.

Table S8: Sensitivity analysis for the association between the dietary contribution of foods groups that take into account both the plant or animal origin of foods and food processing categories, and fatal and non-fatal cardiovascular events, considering the dietary contribution of total grams.

Table S9: Sensitivity analysis for the association between the dietary contribution of food groups that take into account both the red meat or non-red meat origin of foods and food processing categories and fatal and non-fatal cardiovascular events, considering the dietary contribution of total grams.

Table S10: Sensitivity analysis for the association between the dietary contribution of foods groups that take into account both the plant or animal origin of foods and food processing categories, and fatal and non-fatal cardiovascular events, excluded participants with follow-up time <2 years.

Table S11: Sensitivity analysis for the association between the dietary contribution of food groups that take into account both the red meat or non-red meat origin of foods and food processing categories and fatal and non-fatal cardiovascular events from in the UK Biobank cohort, excluded participants with follow-up time <2 years.

Figure S2. Kaplan-Meier pots

| **S1 Table. Examples of food items considered in each food group.** | |
| --- | --- |
| **Plant-sourced foods** | |
| **Non-ultra-processed food** | |
| Fruit | Fresh, squeezed, chilled, frozen, or dried fruits (e.g., banana, orange, raisins); stewed/cooked fruit (e.g. apple, rhubarb, plums); Fruit juice fresh, smoothies, UHT or pasteurised. |
| Beer and Wine | Beer, lager, or cider; Red, rose or white wine (include sparkling). |
| Cereals | Grains such as brown, parboiled, or white rice, couscous, oat and other cooked grains such as bulgur wheat, millet or pearl barley |
| Vegetables | Fresh, chilled or frozen vegetables (e.g. broccoli, cabbage, carrots, cucumber, lettuce, spinach, tomatoes). |
| Pasta | White and wholemeal pasta; Noodles. |
| Roots and tubers | Starchy roots and tubers such as potatoes, sweet potatoes, and cassava; |
| Processed bread | Baguette, ciabatta, paninil; Oat cakes. |
| Nuts and seeds | Peanuts; unsalted, roasted or salted nuts (e.g. almonds, cashews, walnuts); Seeds (e.g. sunflower, pumpkin, linseeds) |
| Table sugar | Sugar; Honey; Syrup. |
| Vegetables/fruit preserved | Olives; Pickle; Sweetcorn (tinned); Jam. |
| Legumes | Beans (kidney beans/chickpeas/butter beans etc) or lentils; Hummus. |
| Others | Coffee/tea, Fungi, Soup without meat, vegetarian sushi, Plant oil |
| **Ultra-processed food** |  |
| Industrialised packaged breads | Slice bread, Bread roll, bap, burger bun, hotdog roll, bagel; Crackers, crispbread, rice cakes, corn cakes |
| Pastries, buns, and cakes | Double or single crust pie/flan; Pancake, crêpe; Yorkshire pudding; Croissant; Scone (plain, fruit, cheese); Fruit cake, Cake, muffin, flapjack, brownie; Doughnuts; Sponge pudding; Cheesecake. |
| Biscuits | Chocolate covered biscuits; Chocolate biscuits; Sweet biscuits; Cereal bars manufactured. |
| Margarine and other spreads | Olive based spread; Margarine; Chocolate/nut spread |
| Industrial chips (French fries) | Potatoes (fried, chips, wedges, roast) |
| Confectionery | Chocolate bars; Chocolate sweets; Low sugar / sugar free sweets (hard and soft); Sweets (hard and soft, e.g. peppermints, toffees, fudge, fruit flavoured sweets) |
| Breakfast cereals | Sweetened oat crunch type cereal; extruded plain cereals with sugars. |
| Soft drinks, fruit drinks, and fruit juices | Fruit drinks and fruit juices, soft drinks, other beverages, Low calories drinks (soft or fruit drinks) |
| Packaged salty snacks | Crisps; Savoury crispbread/corn cake snacks; Cheesy biscuits. |
| Industrial pizza | All type. |
| Packaged pre-prepared meals | Dried/powdered soup; Carton/pouch/canned soup (pea, bean, lentil, vegetables, pasta); Snack pot, noodles/rice; baked beans. |
| Alcoholic drink | Spirits (e.g. vodka, whisky, gin, rum); other alcoholic drinks (e.g. Punch). |
| Sauces, dressing and gravies | Yeast extract; Tomato ketchup; Brown sauce/BBQ sauce; Salad dressing; Tomato-based sauce (e.g. pasta sauce); Gravy. |
| Meat alternatives | Vegetarian sausage/burger; Tofu/tempeh/TVP/soya mince; Quorn |
| **Animal -based foods** | |
| **Non-ultra-processed food** | |
| Red meat | Beef (e.g. roast, steak, mince, curry, burger); Pork (e.g. roast, chops, sweet and sour); Lamb or mutton (e.g. roast, chops, stew, burger); |
| Milk | Cow's milk; Goat's or sheep's milk; power milk; Cream (e.g. single, double, sour, crème fraiche) |
| Fish | Oily fish (e.g. salmon, tinned salmon, herring, mackerel, sardines, fresh tuna steak); White fish (e.g. cod, haddock, fish pie); Prawns, Lobster or crab; Shellfish (e.g. mussels, scallops); Tinned tuna. |
| Cheese | Low fat hard cheese; Hard cheese; Soft cheese; Blue cheese; Cottage cheese; Feta; Mozzarella; Goat's cheese. |
| Poultry | Chicken or turkey (e.g. roast, drumsticks, curry). |
| Animal fats | Butter; Ghee, dripping. |
| Eggs | Whole eggs (e.g. fried, boiled, poached); Omelettes or scrambled egg; Egg in sandwiches; Scotch egg. |
| Milk-based drinks | Dairy/yogurt-based smoothie; Yogurt; Flavoured milk or milkshakes; Hot chocolate (including low calorie). |
| **Ultra-processed food** |  |
| Sausage and other reconstituted red meat products | Sausage; Ham/Parma ham/salami/pastrami/cured meats. |
| Nuggets and other reconstituted meat products | Chicken or turkey in breadcrumbs or deep fried; Liver or liver pâté; Breaded fish (e.g. fish fingers) or fish cakes; Battered fish. |
| Milk based desserts | Ice-cream; Custard, rice pudding; Other milk-based desserts (e.g. mousse, tiramisu, crème caramel) |
| Mayonnaise and spreadable cheese | Mayonnaise/salad cream (including low fat); Cheese sauce (e.g. cauliflower cheese); White sauce/cream sauce (e.g. bechamel) |

| **Table S2. Characteristics of the study population according to quartiles of the dietary contribution of plant-sourced ultra-processed foods, UK Biobank cohort (n =118,397).** | | | | | |
| --- | --- | --- | --- | --- | --- |
|  | **Quartile of the dietary contribution of plant-sourced ultra-processed (% of total energy)** | | | | **P value^*^** |
|  | **1 (22·8%)** | **2 (34·9%)** | **3 (43·6%)** | **4 (56·4%)** |  |
|  | *mean (SD) or % (n)* | | | | |
| **Baseline age, years** | 56·6 (7·5) | 56·4 (7·7) | 55·9 (7·8) | 54·7 (8·1) | *<0·001* |
| **Female sex** | 63·9 (18926) | 58·8 (17390) | 55 (16278) | 50·5 (14957) | *<0·001* |
| **Ethnicity White** | 97·6 (28902) | 97·2 (28779) | 96·9 (28684) | 95·8 (28360) | *<0·001* |
| **Family history of CVD** |  |  |  |  |  |
| No | 44·6 (13201) | 44·6 (13206) | 45·0 (13308) | 46·4 (13732) | *<0·001* |
| Yes, mother or father | 42·3 (12534) | 42·0 (12429) | 42·4 (12542) | 41·7 (12339) |  |
| Yes, mother and father | 13·1 (3865) | 13·4 (3964) | 12·7 (3749) | 11·9 (3528) |  |
| **Baseline BMI status, kg/m^2^** | 26·3 (4·4) | 26·5 (4·4) | 26·7 (4·5) | 27·0 (4·8) | *<0·001* |
| **Pre-existing type 2 diabetes** | 2·8 (815) | 3·0 (884) | 3·6 (1072) | 4·1 (1201) | *<0·001* |
| **Pre-existing high blood pressure** | 21·6 (6386) | 22·3 (6585) | 22·6 (6675) | 229 (6782) | *0.001* |
| **Physical activity** |  |  |  |  |  |
| Low | 13·2 (3910) | 14·9 (4409) | 16·3 (4827) | 18·4 (5434) | *<0·001* |
| Moderate | 37·0 (10940) | 37·8 (11195) | 37·2 (11020) | 36·4 (10759) |  |
| High | 37·1 (10968) | 33·5 (9925) | 31·9 (9441) | 30·3 (8955) |  |
| Missing | 12·8 (3782) | 13·8 (4070) | 14·6 (4311) | 15·0 (4451) |  |
| **Smoking status** |  |  |  |  |  |
| Never smoked | 54·6 (16150) | 57·4 (16976) | 59·2 (17534) | 61·0 (18057) | *<0·001* |
| Ex-smoker | 38·9 (11501) | 36·2 (10703) | 34·1 (10098) | 31·5 (9314) |  |
| Current smoker | 6·6 (1949) | 6·5 (1920) | 6·7 (1967) | 7·5 (2228) |  |
| **Index of Multiple Deprivation** |  |  |  |  |  |
| 1st quintile (least deprived) | 20·3 (6015) | 20·8 (6162) | 20·1 (5947) | 17·8 (5263) | *<0·001* |
| 2nd quintile | 20·3 (5993) | 20·3 (6020) | 19·5 (5785) | 17·9 (5301) |  |
| 3rd quintile | 19·3 (5714) | 19·8 (5869) | 20·0 (5904) | 19·2 (5672) |  |
| 4th quintile | 19·2 (5692) | 18·9 (5601) | 19·3 (5711) | 20·2 (5971) |  |
| 5th quintile (most deprived) | 18·2 (5376) | 17·6 (5200) | 18·7 (5525) | 22·5 (6667) |  |
| Missing | 2·7 (810) | 2·5 (747) | 2·5 (727) | 2·5 (725) |  |
| **Geographical region** |  |  |  |  |  |
| London | 26·8 (7933) | 21·6 (6393) | 18·4 (5444) | 15·5 (4599) | *<0·001* |
| South East | 10·2 (3014) | 9·7 (2879) | 9·2 (2734) | 8·3 (2464) |  |
| South West | 10·4 (3088) | 10·7 (3160) | 10·7 (3167) | 9·8 (2907) |  |
| East Midlands | 5·3 (1578) | 6·1 (1803) | 6·7 (1974) | 6·6 (1964) |  |
| West Midlands | 6·4 (1904) | 7·7 (2277) | 8 (2381) | 9·4 (2786) |  |
| Yorkshire & the Humber | 13·7 (4042) | 15·3 (4541) | 15·9 (4706) | 16·5 (4892) |  |
| North East | 7·7 (2289) | 8·5 (2508) | 10·0 (2960) | 11·5 (3417) |  |
| North West | 11·3 (3350) | 12·1 (3585) | 12·3 (3645) | 13·4 (3952) |  |
| Wales | 2·9 (847) | 2·9 (862) | 3·3 (982) | 3·4 (1010) |  |
| Scotland | 5·3 (1555) | 5·4 (1591) | 5·4 (1606) | 5·4 (1608) |  |
| **Nutrients** |  |  |  |  |  |
| Total energy (kcal) | 1896 (522) | 2008 (512) | 2071 (521) | 2160 (562) | *<0·001* |
| Free sugars (% of energy) | 12·1 (7·1) | 13·2 (6·3) | 13·9 (5·9) | 15·0 (6·2) | *<0·001* |
| Saturated fats (% of energy) | 10·4 (3·2) | 10·9 (3·0) | 11·1 (3·0) | 11·3 (2·9) | *<0·001* |
| Fibre (g/1000 kcal) | 13·1 (4·8) | 12·6 (4·3) | 12·4 (4·2) | 12·2 (4·3) | *<0·001* |
| Sodium (mg/1000 kcal) | 853 (216) | 915 (200) | 957 (203) | 1015 (218) | *<0·001* |
| BMI = Body Mass Index. * Analysis of variance or χ2 test where appropriate. | | | | | |
|  | | |  |  |  |

| **Table S3. Test for linearity assumption of association between the food groups and fatal and non-fatal cardiovascular events, using restricted cubic spline functions.** | | | | | | | |
| --- | --- | --- | --- | --- | --- | --- | --- |
|  | **Incidence** | | |  | **Mortality** | | |
|  | All cardiovascular diseases | Coronary heart disease | Cerebrovascular disease |  | All cardiovascular diseases | Coronary heart disease | Cerebrovascular disease |
|  | *P value* | | | | | | |
| Plant-sourced non-UPF | 0·264 | 0·408 | 0·275 |  | 0·213 | 0·399 | 0·616 |
| Plant-sourced UPF | 0·360 | 0·581 | 0·708 |  | 0·468 | 0·242 | 0·061 |
| All plant-sourced foods | 0·116 | 0·185 | 0·422 |  | 0·039 | 0·026 | 0·484 |
| All UPF | 0·571 | 0·880 | 0·943 |  | 0·415 | 0·505 | 0·178 |
| Non-red meat non-UPF | 0·862 | 0·814 | 0·949 |  | 0·536 | 0·344 | 0·459 |
| Non-red meat UPF | 0·445 | 0·539 | 0·739 |  | 0·972 | 0·766 | 0·760 |
| All non-red meat | 0·979 | 0·927 | 0·988 |  | 0·128 | 0·343 | 0·202 |
| Animal-sourced non-UPF | 0·117 | 0·063 | 0·929 |  | 0·467 | 0·505 | 0·755 |
| Animal-sourced UPF | 0·716 | 0·538 | 0·700 |  | 0·160 | 0·031 | 0·509 |
| All animal-sourced foods | 0·966 | 0·881 | 0·828 |  | 0·879 | 0·968 | 0·925 |
| UPF = Ultra-processed foods. | | | | | | | |

| **Table S4. Association between the dietary contribution of food groups that take into account both the red meat or non-red meat origin of foods and food processing categories and fatal and non-fatal cardiovascular events from in the UK Biobank cohort (n = 118,397).** | | | | | | |
| --- | --- | --- | --- | --- | --- | --- |
|  | **Dietary contribution (% of total energy)** | | | | | |
| **Food groups** | **Q1** | **Q2** | **Q3** | **Q4** |  | **Continuous (10% increase in the contribution)** |
|  | *HR (95% CI)* | | | | *p for trend* | *HR (95% CI)* |
|  | **All cardiovascular diseases** | | | | | |
|  | *n for cases/non-cases = 7,806/110,591* | | | | | |
| ***Non-red meat non-UPF*** | 1 | 0·94 (0·88–1.00) | 0·89 (0·83–0·95) | 0·81 (0·75–0·86) | <0·001 | 0·94 (0·93–0·96) |
| ***Non-red meat UPF*** | 1 | 1·11 (1·04–1·19) | 1·16 (1·08–1·23) | 1·25 (1·17–1·34) | <0·001 | 1·06 (1·04–1·08) |
| ***All non-red meat*** | 1 | 1·01 (0·95–1·07) | 0·99 (0·93–1·06) | 0·97 (0·91–1·03) | 0·331 | 0·99 (0·95–1·03) |
|  | **Coronary heart disease** | | | | | |
|  | *n for cases/non-cases =6,006/112,391* | | | | | |
| ***Non-red meat non-UPF*** | 1 | 0·95 (0·89–1·02) | 0·89 (0·83–0·96) | 0·76 (0·71–0·82) | *<0·001* | 0·93 (0·91–0·95) |
| ***Non-red meat UPF*** | 1 | 1·18 (1·09–1·27) | 1·25 (1·16–1·34) | 1·31 (1·22–1·42) | *<0·001* | 1·07 (1·05–1·10) |
| ***All non-red meat*** | 1 | 1·01 (0·94–1·08) | 0·98 (0·91–1·05) | 0·96 (0·89–1·04) | *0·244* | 0·98 (0·94–1·03) |
|  | Cerebrovascular disease | | | | | |
|  | *n for cases/non-cases = 2,112/116,285* | | | | | |
| ***Non-red meat non-UPF*** | 1 | 0·92 (0·82–1·04) | 0·9 (0·79–1·01) | 0·99 (0·88–1·12) | 0·793 | 0·99 (0·96–1·03) |
| ***Non-red meat UPF*** | 1 | 0·93 (0·82–1·05) | 0·92 (0·81–1·04) | 1·07 (0·95–1·21) | 0·309 | 1·01 (0·98–1·05) |
| ***All non-red meat*** | 1 | 1·03 (0·91–1·16) | 1·05 (0·93–1·18) | 1·02 (0·9–1·16) | 0·636 | 1·04 (0·96–1·12) |
|  | **All cardiovascular diseases mortality** | | | | | |
|  | *n for cases /non-cases = 529/117,868* | | | | | |
| ***Non-red meat non-UPF*** | 1 | 0·95 (0·76–1·19) | 0·82 (0·65–1·04) | 0·69 (0·53–0·89) | 0·002 | 0·91 (0·85–0·97) |
| ***Non-red meat UPF*** | 1 | 1·14 (0·88–1·47) | 1·18 (0·92–1·52) | 1·38 (1·08–1·77) | 0·012 | 1·09 (1·02–1·16) |
| ***All non-red meat*** | 1 | 0·84 (0·67–1·06) | 0·84 (0·67–1·07) | 0·92 (0·72–1·18) | 0·447 | 0·93 (0·80–1·01) |
|  | **Coronary heart disease mortality** | | | | | |
|  | *n for cases/non-cases = 348/118,049* | | | | | |
| ***Non-red meat non-UPF*** | 1 | 0·95 (0·73–1·25) | 0·87 (0·65–1·16) | 0·57 (0·41–0·79) | *0·001* | 0·87 (0·80–0·94) |
| ***Non-red meat UPF*** | 1 | 1·35 (0·97–1·88) | 1·56 (1·14–2·15) | 1·56 (1·13–2·15) | *0·005* | 1·13 (1·05–1·22) |
| ***All non-red meat*** | 1 | 0·89 (0·67–1·17) | 0·77 (0·57–1·03) | 0·85 (0·62–1·15) | *0·152* | 0·90 (0·75–1·07) |
|  | **Cerebrovascular disease mortality** | | | | | |
|  | *n for cases/non-cases = 181/118,216* | | | | | |
| ***Non-red meat non-UPF*** | 1 | 0·95 (0·64–1·41) | 0·73 (0·48–1·12) | 0·91 (0·61–1·38) | 0·430 | . |
| ***Non-red meat UPF*** | 1 | 0·88 (0·58–1·32) | 0·69 (0·45–1·08) | 1·17 (0·79–1·73) | 0·644 | 1·00 (0·90–1·12) |
| ***All non-red meat*** | 1 | 0·75 (0·49–1·15) | 1·01 (0·68–1·5) | 1·07 (0·71–1·61) | 0·487 | 1·01 (0·78–1·32) |
| UPF = ultra-processed foods | | | | | | |
| Mean follow-up times were 9·1 for overall cardiovascular disease (1,076,104 person-years), 9·2 coronary heart disease (1,083,490 person-years), and 9·3 for cerebrovascular diseases (1,101,715 person-years). Mean follow-up times were 9·2 for mortality for cardiovascular disease (1,091,678 person-years), coronary heart disease (1,091,678 person-years), and cerebrovascular diseases (1,091,678 person-years). | | | | | | |
| Cut-off for quarters of food contribution ranged from 86·0% of total energy intake (1st quartile) to 99·9% (4th quartile) for non-red meat; from 29·7% to 65·4% for non-red meat non-UPF; and from 29·1% to 64·2% for non-red meat UPF, respectively. | | | | | | |
| Cox proportional hazards models with age as the underlying timescale. Adjusted by sex, ethnic (white, non-white), family history of CVD (no, mother or father, mother and father), BMI (continuous), physical activity (low, moderate, high, missing), smoking status (never, previous, current), index of multiple deprivation (quintile), and region (London, South East, South West, East Midlands, West Midlands, Yorkshire & the Humber, North East, North West, Wales, Scotland). Analysis for risk of CVD were stratified by sex, family history of CVD and smoking status. Analysis for the CVD death were stratified by sex and ethnic. | | | | | | |

**
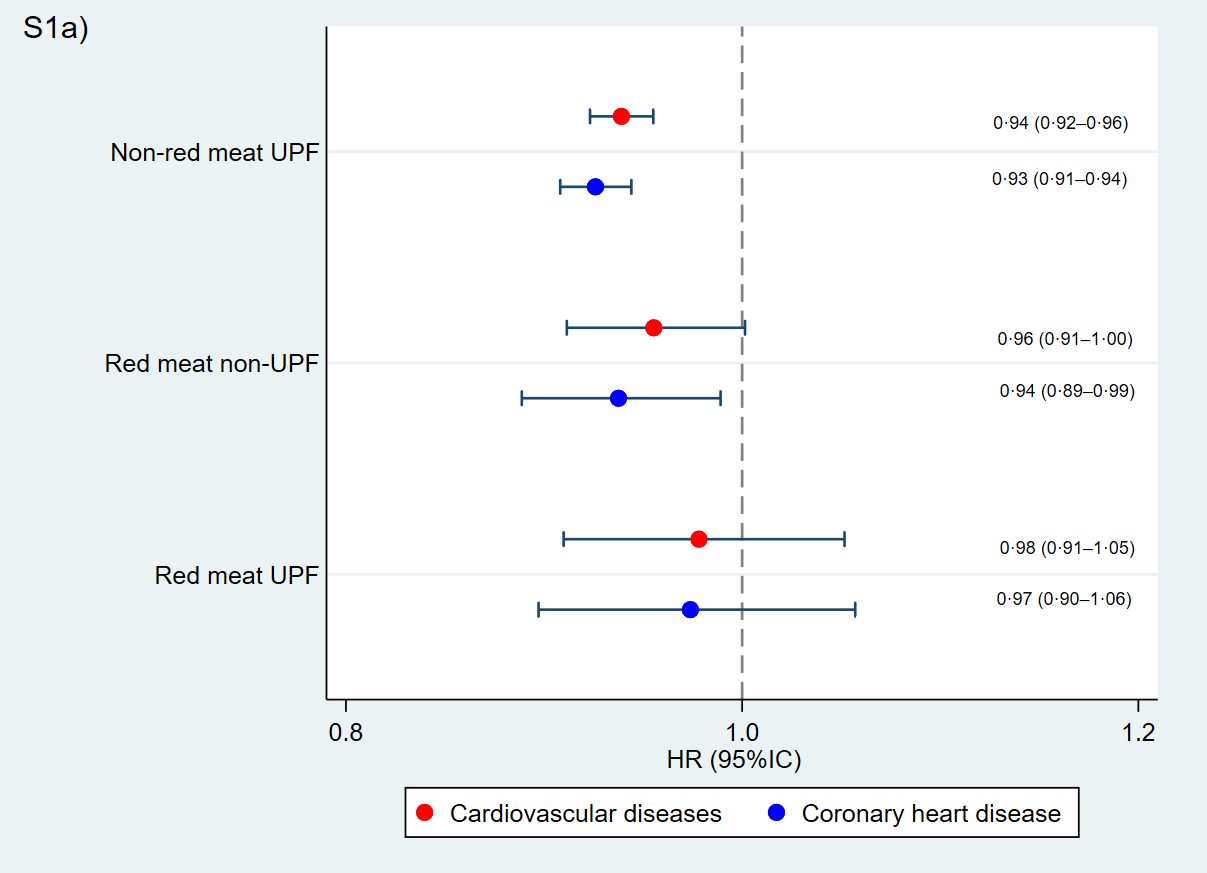

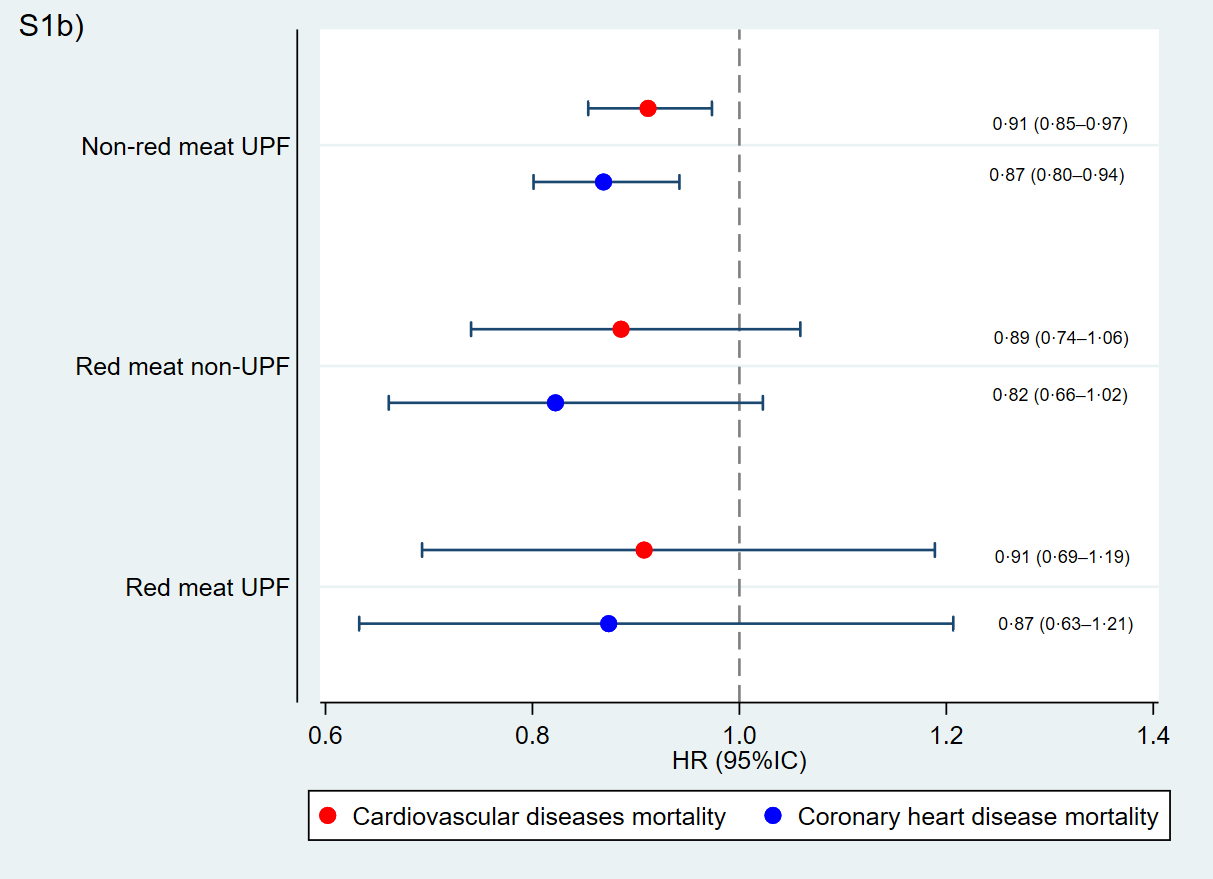
**

**Figure S1. Effect of replacing 10% of each of the 3 food groups (non-red meat UPF, red meat non-UPF, and red meat UPF) with non-red meat non-UPF**

Note: Food substitutions among UK Biobank participants (n = 118,397). Fully adjusted hazard ratios (HR) and 95% confidence intervals (CI) were calculated using Cox proportional hazards regression to assess the substitutions of contributions from food groups that take into account both the red meat or non-red meat origin of foods as well as food processing categories, and their association with cardiovascular incidence (Figure S1a) and cardiovascular mortality (Figure S1b), using Cox proportional hazards regression. All results are from continuous linear models. Non-red meat non-ultra-processed (non-Meat non-UPF) by non-red meat ultra-processed, red meat ultra-processed or red meat non-ultra-processed. Non-red meat ultra-processed by red meat non-ultra-processed or red meat ultra-processed. Red meat non-ultra-processed by red meat ultra-processed. UPF = ultra-processed.

**Concise description of the results from Table S4 and figure S1: Non-red meat intake and cardiovascular disease incidence and mortality**

The analyses of the association between the dietary contribution of food groups that consider both the red meat or non-red meat origin of foods and food processing categories, and fatal and non-fatal cardiovascular events are shown in Table S4. After adjustment for potential confounders, the dietary contribution of non-red meat non-UPF was inversely associated with all CVD (adjusted HR for a 10% increase in the contribution: 0·94; 95% CI 0·93–0·96) and coronary heart disease (adjusted HR 0·93; 95% CI 0·91–0·95) incidence; while non-red meat UPF contribution was associated with increased risk of both outcomes (adjusted HR 1·06; 95% CI 1·04–1·08 for all CVD; and adjusted HR 1·07; 95% CI 1·05–1·10 for coronary heart disease). The contribution of all non-red meat in diet was not associated with any of the CVD events.

Associations observed for mortality were similar to those seen for CVD incidence. The dietary contribution of non-red meat non-UPF was inversely associated with mortality for all CVD (adjusted HR for a 10% increase in the contribution: 0·91; 95% CI 0·85–0·97) and coronary heart disease (adjusted HR 0·87; 95% CI 0·80–0·94); while non-red meat UPF contribution was positively associated with a higher mortality for all CVD (adjusted HR 1·09; 95% CI 1·02–1·16) and coronary heart disease (adjusted HR 1·13; 95% CI 1·05–1·22).

The analyses using quartiles of the dietary contribution showed consistent trends with analysis using continuous variables (per 10% increase in the contribution). There was no evidence of a significant association for any of the food groups and cerebrovascular incidence or mortality.

In our substitution analysis (Figure S1), replacing 10% of non-red meat UPF or red meat non-UPF (only for coronary heart disease) with an equal amount of daily energy from non-red meat non-UPF was associated with a reduced risk of all CVD and coronary heart disease. The findings from the substitution models were consistent in the mortality analysis, with the exception of substituting red meat non-UPF for non-red meat non-UPF that did not reach statistical significance for both outcomes, although the HR indicated some level of protection.

| **Table S5. Association between the dietary contribution of foods groups that take into account both the plant or animal origin of foods and food processing categories, and fatal and non-fatal cardiovascular events from in the UK Biobank cohort, additionally adjustment for animal-sourced ultra-processed foods and for red meat ultra-processed foods (n = 118,397).** | | | | | | |
| --- | --- | --- | --- | --- | --- | --- |
|  | **Dietary contribution (% of total energy)** | | | | | |
| **Food groups** | **Q1** | **Q2** | **Q3** | **Q4** |  | **Continuous (10% increase in the contribution)** |
|  | *HR (95% CI)* | | | | *p for trend* | *HR (95% CI)* |
|  | **All cardiovascular diseases** | | | | | |
| ***Animal-sourced non-UPF*** | 1 | 0·92 (0·86–0·98) | 0·94 (0·88–1·00) | 0·93 (0·87–0·99) | *0·058* | 0·98 (0·95–1·00) |
| ***Animal-sourced UPF*** | 1 | 1·00 (0·94–1·07) | 1·00 (0·94–1·06) | 1·07 (1·00–1·14) | *0·047* | 1·04 (1·01–1·07) |
| ***All animal-sourced foods*** | 1 | 0·97 (0·91–1·04) | 1·02 (0·95–1·08) | 1·03 (0·96–1·09) | *0·228* | 1·01 (0·99–1·03) |
| ***Plant-sourced non-UPF*** |  |  |  |  |  |  |
| *Model + animal-sourced UPF* | 1 | 0·90 (0·84–0·95) | 0·86 (0·8–0·91) | 0·81 (0·76–0·87) | *<0·001* | 0·94 (0·92–0·95) |
| *Model + red meat UPF* | 1 | 0·89 (0·84–0·95) | 0·85 (0·8–0·91) | 0·80 (0·75–0·86) | *<0·001* | 0·93 (0·91–0·95) |
| ***Plant-sourced UPF*** |  |  |  |  |  |  |
| *Model + animal-sourced UPF* | 1 | 1·06 (0·99–1·13) | 1·16 (1·09–1·24) | 1·19 (1·12–1·27) | *<0·001* | 1·06 (1·04–1·08) |
| *Model + red meat UPF* | 1 | 1·05 (0·98–1·12) | 1·15 (1·07–1·22) | 1·16 (1·09–1·24) | *<0·001* | 1·05 (1·03–1·07) |
|  | **Coronary heart disease** | | | | | |
| ***Animal-sourced non-UPF*** | 1 | 0·90 (0·84–0·97) | 0·94 (0·87–1·00) | 0·93 (0·87–1·00) | *0·115* | 0·97 (0·95–1·00) |
| ***Animal-sourced UPF*** | 1 | 1·02 (0·95–1·10) | 1·03 (0·96–1·11) | 1·10 (1·03–1·18) | *0·009* | 1·04 (1·01–1·08) |
| ***All animal-sourced foods*** | 1 | 0·98 (0·91–1·06) | 1·04 (0·97–1·12) | 1·03 (0·95–1·10) | *0·242* | 1·01 (0·98–1·04) |
| ***Plant-sourced non-UPF*** |  |  |  |  |  |  |
| *Model + animal-sourced UPF* | 1 | 0·89 (0·83–0·96) | 0·84 (0·78–0·9) | 0·78 (0·72–0·84) | *<0·001* | 0·92 (0·90–0·94) |
| *Model + red meat UPF* | 1 | 0·89 (0·83–0·95) | 0·84 (0·78–0·9) | 0·77 (0·71–0·83) | *<0·001* | 0·92 (0·89–0·94) |
| ***Plant-sourced UPF*** |  |  |  |  |  |  |
| *Model + animal-sourced UPF* | 1 | 1·10 (1·02–1·18) | 1·22 (1·13–1·32) | 1·25 (1·16–1·34) | *<0·001* | 1·07 (1·05–1·10) |
| *Model + red meat UPF* | 1 | 1·09 (1·01–1·17) | 1·20 (1·12–1·29) | 1·21 (1·13–1·31) | *<0·001* | 1·06 (1·04–1·09) |
|  | **Cerebrovascular disease** | | | | | |
| ***Animal-sourced non-UPF*** | 1 | 0·97 (0·86–1·10) | 1·00 (0·88–1·12) | 0·95 (0·84–1·08) | *0·526* | 0·99 (0·94–1·05) |
| ***Animal-sourced UPF*** | 1 | 0·94 (0·83–1·06) | 0·91 (0·81–1·03) | 0·95 (0·84–1·07) | *0·321* | 1·00 (0·95–1·06) |
| ***All animal-sourced foods*** | 1 | 0·98 (0·86–1·10) | 0·95 (0·84–1·07) | 1·03 (0·91–1·16) | *0·726* | 1·00 (0·96–1·04) |
| ***Plant-sourced non-UPF*** |  |  |  |  |  |  |
| *Model + animal-sourced UPF* | 1 | 0·91 (0·81–1·03) | 0·93 (0·82–1·05) | 0·93 (0·81–1·05) | *0·306* | 0·99(0·95–1·03) |
| *Model + red meat UPF* | 1 | 0·91 (0·81–1·03) | 0·93 (0·83–1·05) | 0·93 (0·82–1·05) | *0·288* | 0·99 (0·95–1·03) |
| ***Plant-sourced UPF*** |  |  |  |  |  |  |
| *Model + animal-sourced UPF* | 1 | 0·94 (0·84–1·07) | 0·97 (0·86–1·09) | 1·03 (0·91–1·17) | *0·580* | 1·01 (0·98–1·05) |
| *Model + red meat UPF* | 1 | 0·94 (0·84–1·06) | 0·96 (0·85–1·09) | 1·03 (0·91–1·16) | *0·609* | 1·01 (0·98–1·05) |
|  | **All cardiovascular diseases mortality** | | | | | |
| ***Animal-sourced non-UPF*** | 1 | 0·79 (0·61–1·02) | 1·00 (0·79–1·26) | 1·05 (0·83–1·33) | *0·336* | 1·04 (0·94–1·15) |
| ***Animal-sourced UPF*** | 1 | 0·94 (0·74–1·20) | 1·10 (0·87–1·40) | 0·93 (0·73–1·19) | *0·920* | 0·96 (0·86–1·07) |
| ***All animal-sourced foods*** | 1 | 1·00 (0·77–1·28) | 1·18 (0·93–1·51) | 1·12 (0·87–1·44) | *0·195* | 1·00 (0·92–1·09) |
| ***Plant-sourced non-UPF*** |  |  |  |  |  |  |
| *Model + animal-sourced UPF* | 1 | 0·88 (0·70–1·10) | 0·75 (0·59–0·95) | 0·58 (0·44–0·75) | *<0·001* | 0·85 (0·78–0·92) |
| *Model + red meat UPF* | 1 | 0·90 (0·72–1·13) | 0·78 (0·62–0·99) | 0·61 (0·47–0·79) | *<0·001* | 0·87 (0·80–0·94) |
| ***Plant-sourced UPF*** |  |  |  |  |  |  |
| *Model + animal-sourced UPF* | 1 | 1·00 (0·77–1·31) | 1·39 (1·08–1·78) | 1·49 (1·15–1·92) | *<0·001* | 1·12 (1·04–1·20) |
| *Model + red meat UPF* | 1 | 1·00 (0·77–1·31) | 1·39 (1·08–1·78) | 1·49 (1·16–1·92) | *<0·001* | 1·12 (1·05–1·20) |
|  | **Coronary heart disease mortality** | | | | | |
| ***Animal-sourced non-UPF*** | 1 | 0·82 (0·60–1·12) | 1·08 (0·81–1·45) | 1·18 (0·88–1·58) | *0·106* | 1·07 (0·95–1·22) |
| ***Animal-sourced UPF*** | 1 | 0·96 (0·71–1·29) | 1·13 (0·85–1·50) | 0·89 (0·66–1·21) | *0·759* | 0·93 (0·81–1·07)^*^ |
| ***All animal-sourced foods*** | 1 | 1·01 (0·74–1·38) | 1·27 (0·95–1·71) | 1·17 (0·86–1·59) | *0·151* | 1·01 (0·91–1·12) |
| ***Plant-sourced non-UPF*** |  |  |  |  |  |  |
| *Model + animal-sourced UPF* | 1 | 0·81 (0·61–1·06) | 0·67 (0·50–0·89) | 0·44 (0·31–0·61) | *<0·001* | 0·77 (0·70–0·85) |
| *Model + red meat UPF* | 1 | 0·84 (0·64–1·10) | 0·71 (0·53–0·94) | 0·48 (0·34–0·66) | *<0·001* | 0·80 (0·72–0·88) |
| ***Plant-sourced UPF*** |  |  |  |  |  |  |
| *Model + animal-sourced UPF* | 1 | 1·32 (0·93–1·87) | 1·75 (1·25–2·44) | 1·89 (1·35–2·64) | *<0·001* | 1·18 (1·09–1·28) |
| *Model + red meat UPF* | 1 | 1·32 (0·93–1·87) | 1·75 (1·26–2·44) | 1·91 (1·37–2·65) | *<0·001* | 1·18 (1·09–1·29) |
|  | **Cerebrovascular disease mortality** | | | | | |
| ***Animal-sourced non-UPF*** | 1 | 0·74 (0·48–1·13) | 0·84 (0·56–1·26) | 0·83 (0·56–1·25) | *0·524* | 0·97 (0·81–1·15) |
| ***Animal-sourced UPF*** | 1 | 0·92 (0·60–1·41) | 1·06 (0·70–1·6) | 1·02 (0·67–1·54) | *0·784* | 1·01 (0·85–1·20) |
| ***All animal-sourced foods*** | 1 | 0·96 (0·62–1·48) | 1·02 (0·66–1·55) | 1·03 (0·67–1·57) | *0·827* | 0·98 (0·85–1·14) |
| ***Plant-sourced non-UPF*** |  |  |  |  |  |  |
| *Model + animal-sourced UPF* | 1 | 1·06 (0·71–1·59) | 0·97 (0·63–1·49) | 0·95 (0·61–1·49) | *0·747* | 1·01 (0·88–1·16) |
| *Model + red meat UPF* | 1 | 1·06 (0·71–1·59) | 0·97 (0·64–1·48) | 0·96 (0·62–1·47) | *0·745* | 1·01 (0·88–1·15) |
| ***Plant-sourced UPF*** |  |  |  |  |  |  |
| *Model + animal-sourced UPF* | 1 | 0·67 (0·43–1·04) | 1·01 (0·68–1·50) | 1·06 (0·70–1·60) | *0·453* | 1·00 (0·89–1·13) |
| *Model + red meat UPF* | 1 | 0·67 (0·43–1·04) | 1·00 (0·67–1·49) | 1·05 (0·70–1·57) | *0·471* | 1·00 (0·89–1·13) |
| Q = Quartile. UPF = Ultra-processed foods. ^*^Non-linear association in restricted cubic spline regression (p=0·03). | | | | | | |
| Cox proportional hazards models with age as the underlying timescale. Adjusted by sex, ethnic (white, non-white), family history of CVD (no, mother or father, mother and father), BMI (continuous), physical activity (low, moderate, high, missing), smoking status (never, previous, current), index of multiple deprivation (quintile), and region (London, South East, South West, East Midlands, West Midlands, Yorkshire & the Humber, North East, North West, Wales, Scotland). Analysis for risk of CVD were stratified by sex, family history of CVD and smoking status. Analysis for the CVD death were stratified by sex and ethnic. Model was additionally adjusted for animal-sourced UPF and for red meat UPF. | | | | | | |

| **Table S6. Association between the dietary contribution of foods groups that take into account both the plant or animal origin of foods and food processing categories, and fatal and non-fatal cardiovascular events from in the UK Biobank cohort, additionally adjustment for alcohol, nutrients and baseline status for type 2 diabetes and high blood pressure (n = 118,397)** | | | | | | |
| --- | --- | --- | --- | --- | --- | --- |
|  | **Dietary contribution (% of total energy)** | | | | | |
| **Food groups** | **Q1** | **Q2** | **Q3** | **Q4** |  | **Continuous (10% increase in the contribution)** |
|  | *HR (95% CI)* | | | | *p for trend* | *HR (95% CI)* |
|  | **All cardiovascular diseases** | | | | | |
| ***Plant-sourced non-UPF*** |  |  |  |  |  |  |
| *Model + alcohol* | 1 | 0·90 (0·84–0·95) | 0·86 (0·80–0·91) | 0·81 (0·76–0·87) | *<0·001* | 0·94 (0·92–0·96) |
| *Model + nutrients* | 1 | 0·90 (0·85–0·96) | 0·86 (0·81–0·92) | 0·81 (0·75–0·88) | *<0·001* | 0·93 (0·91–0·96) |
| *Model + baseline DM/HBP* | 1 | 0·90 (0·85–0·96) | 0·86 (0·81–0·92) | 0·81 (0·76–0·87) | *<0·001* | 0·94 (0·92–0·96) |
| ***Plant-sourced UPF*** |  |  |  |  |  |  |
| *Model + alcohol* | 1 | 1·04 (0·98–1·11) | 1·14 (1·06–1·21) | 1·15 (1·07–1·22) | *<0·001* | 1·05 (1·03–1·07) |
| *Model + nutrients* | 1 | 1·03 (0·97–1·10) | 1·12 (1·04–1·19) | 1·11 (1·04–1·19) | *<0·001* | 1·04 (1·02–1·06) |
| *Model + baseline DM/HBP* | 1 | 1·05 (0·98–1·12) | 1·14 (1·07–1·21) | 1·15 (1·08–1·23) | *<0·001* | 1·05 (1·03–1·07) |
|  | **Coronary heart disease** | | | | | |
| ***Plant-sourced non-UPF*** |  |  |  |  |  |  |
| *Model + alcohol* | 1 | 0·90 (0·84–0·96) | 0·85 (0·79–0·91) | 0·79 (0·73–0·85) | *<0·001* | 0·92 (0·90–0·95) |
| *Model + nutrients* | 1 | 0·89 (0·83–0·96) | 0·84 (0·78–0·90) | 0·76 (0·70–0·83) | *<0·001* | 0·91 (0·88–0·93) |
| *Model + baseline DM/HBP* | 1 | 0·90 (0·84–0·96) | 0·85 (0·79–0·91) | 0·78 (0·72–0·84) | *<0·001* | 0·92 (0·90–0·94) |
| ***Plant-sourced UPF*** |  |  |  |  |  |  |
| *Model + alcohol* | 1 | 1·08 (1·00–1·17) | 1·18 (1·10–1·27) | 1·18 (1·10–1·28) | *<0·001* | 1·06 (1·04–1·08) |
| *Model + nutrients* | 1 | 1·07 (0·99–1·16) | 1·17 (1·08–1·26) | 1·15 (1·07–1·25) | *<0·001* | 1·05 (1·03–1·07) |
| *Model + baseline DM/HBP* | 1 | 1·09 (1·01–1·17) | 1·19 (1·11–1·28) | 1·20 (1·11–1·29) | *<0·001* | 1·06 (1·04–1·08) |
|  | **Cerebrovascular disease** | | | | | |
| ***Plant-sourced non-UPF*** |  |  |  |  |  |  |
| *Model + alcohol* | 1 | 0·91 (0·80–1·02) | 0·92 (0·81–1·04) | 0·91 (0·80–1·03) | *0·190* | 0·98 (0·94–1·04) |
| *Model + nutrients* | 1 | 0·95 (0·84–1·07) | 0·99 (0·87–1·13) | 1·02 (0·88–1·18) | *0·645* | 1·03 (0·98–1·08) |
| *Model + baseline DM/HBP* | 1 | 0·93 (0·82–1·04) | 0·94 (0·84–1·07) | 0·94 (0·83–1·06) | 0·406 | 0·99 (0·96–1·03) |
| ***Plant-sourced UPF*** |  |  |  |  |  |  |
| *Model + alcohol* | 1 | 0·95 (0·84–1·07) | 0·97 (0·86–1·10) | 1·04 (0·92–1·18) | *0·507* | 1·01 (0·98–1·05) |
| *Model + nutrients* | 1 | 0·93 (0·82–1·05) | 0·94 (0·83–1·07) | 1·00 (0·88–1·14) | *0·961* | 1·00 (0·97–1·04) |
| *Model + baseline DM/HBP* | 1 | 0·94 (0·83–1·06) | 0·96 (0·85–1·08) | 1·01 (0·90–1·15) | 0·779 | 1·01 (0·97–1·04) |
|  | **All cardiovascular diseases mortality** | | | | | |
| ***Plant-sourced non-UPF*** |  |  |  |  |  |  |
| *Model + alcohol* | 1 | 0·90 (0·71–1·12) | 0·77 (0·61–0·99) | 0·6 (0·46–0·79) | *<0·001* | 0·86 (0·79–0·94) |
| *Model + nutrients* | 1 | 0·89 (0·71–1·12) | 0·77 (0·60–0·99) | 0·59 (0·44–0·80) | *<0·001* | 0·86 (0·78–0·94) |
| *Model + baseline DM/HBP* | 1 | 0·91 (0·72–1·14) | 0·8 (0·63–1·02) | 0·63 (0·49–0·82) | *<0·001* | 0·88 (0·81–0·95) |
| ***Plant-sourced UPF*** |  |  |  |  |  |  |
| *Model + alcohol* | 1 | 1·00 (0·77–1·31) | 1·38 (1·07–1·78) | 1·48 (1·14–1·91) | *<0·001* | 1·11 (1·04–1·19) |
| *Model + nutrients* | 1 | 0·99 (0·76–1·30) | 1·36 (1·05–1·75) | 1·45 (1·11–1·88) | *<0·001* | 1·11 (1·03–1·19) |
| *Model + baseline DM/HBP* | 1 | 0·99 (0·75–1·29) | 1·36 (1·06–1·75) | 1·45 (1·13–1·86) | *<0·001* | 1·11 (1·04–1·19) |
|  | **Coronary heart disease mortality** | | | | | |
| ***Plant-sourced non-UPF*** |  |  |  |  |  |  |
| *Model + alcohol* | 1 | 0·82 (0·62–1·08) | 0·68 (0·51–0·92) | 0·45 (0·31–0·64) | *<0·001* | 0·78 (0·70–0·87) |
| *Model + nutrients* | 1 | 0·85 (0·64–1·12) | 0·72 (0·53–0·98) | 0·49 (0·33–0·72) | *<0·001* | 0·90 (0·71–0·90) |
| *Model + baseline DM/HBP* | 1 | 0·84 (0·64–1·10) | 0·73 (0·55–0·97) | 0·49 (0·36–0·69) | *<0·001* | 0·81 (0·74–0·89) |
| ***Plant-sourced UPF*** |  |  |  |  |  |  |
| *Model + Alcohol* | 1 | 1·32 (0·93–1·87) | 1·76 (1·26–2·45) | 1·91 (1·36–2·68) | *<0·001* | 1·18 (1·09–1·29) |
| *Model + Nutrients* | 1 | 1·28 (0·9–1·81) | 1·67 (1·19–2·34) | 1·79 (1·27–2·52) | *<0·001* | 1·16 (1·06–1·27) |
| *Model + baseline DM/HBP* | 1 | 1·29 (0·91–1·83) | 1·72 (1·23–2·40) | 1·84 (1·32–2·56) | *<0·001* | 1·17 (1·08–1·27) |
|  | **Cerebrovascular disease mortality** | | | | | |
| ***Plant-sourced non-UPF*** |  |  |  |  |  |  |
| *Model + alcohol* | 1 | 1·08 (0·72–1·63) | 1·01 (0·66–1·55) | 1·01 (0·65–1·6) | *0·971* | 1·04 (0·90–1·19) |
| *Model + nutrients* | 1 | 1·00 (0·66–1·52) | 0·88 (0·56–1·37) | 0·8 (0·49–1·32) | *0·323* | 0·96 (0·82–1·12) |
| *Model + baseline DM/HBP* | 1 | 1·08 (0·72–1·62) | 0·99 (0·65–1·51) | 0·98 (0·64–1·5) | 0·827 | 1·02 (0·89–1·16) |
| ***Plant-sourced UPF*** |  |  |  |  |  |  |
| *Model + alcohol* | 1 | 0·66 (0·43–1·03) | 0·98 (0·66–1·47) | 1·02 (0·67–1·54) | 0·580 | 0·99 (0·88–1·12) |
| *Model + nutrients* | 1 | 0·69 (0·44–1·07) | 1·04 (0·69–1·56) | 1·10 (0·72–1·68) | *0·361* | 1·01 (0·90–1·15) |
| *Model + baseline DM/HBP* | 1 | 0·67 (0·43–1·03) | 0·99 (0·66–1·47) | 1·03 (0·69–1·54) | 0·535 | 1·00 (0·89–1·12) |
| Q = Quartile. UPF = Ultra-processed foods. DM = Type 2 diabetes mellitus. HBP = High blood pressure. | | | | | | |
| Cox proportional hazards models with age as the underlying timescale. Adjusted by sex, ethnic (white, non-white), family history of CVD (no, mother or father, mother and father), BMI (continuous), physical activity (low, moderate, high, missing), smoking status (never, previous, current), index of multiple deprivation (quintile), and region (London, South East, South West, East Midlands, West Midlands, Yorkshire & the Humber, North East, North West, Wales, Scotland). Analysis for risk of CVD were stratified by sex, family history of CVD and smoking status. Analysis for the CVD death were stratified by sex and ethnic. Model was additionally adjusted for alcohol intake (g/day), nutrients (% free sugar of total energy, % saturated fat of total energy, sodium density and fibre density) and status of type II diabetes mellitus or high blood pressure at baseline. | | | | | | |

| **Table S7. Association between the dietary contribution of food groups that take into account both the red meat or non-red meat origin of foods and food processing categories and fatal and non-fatal cardiovascular events from in the UK Biobank cohort, additionally adjustment for alcohol, nutrients and baseline status for type 2 diabetes and high blood pressure (n = 118,397)** | | | | | | |
| --- | --- | --- | --- | --- | --- | --- |
|  | **Dietary contribution (% of total energy)** | | | | | |
| **Food groups** | **Q1** | **Q2** | **Q3** | **Q4** |  | **Continuous (10% increase in the contribution)** |
|  | *HR (95% CI)* | | | | *p for trend* | *HR (95% CI)* |
|  | **All cardiovascular diseases** | | | | | |
| ***Non-red meat non-UPF*** |  |  |  |  |  |  |
| *Model + alcohol* | 1 | 0·94 (0·89–1·00) | 0·9 (0·84–0·95) | 0·82 (0·76–0·87) | *<0·001* | 0·94 (0·93–0·96) |
| *Model + nutrients* | 1 | 0·97 (0·91–1·03) | 0·93 (0·87–0·99) | 0·86 (0·80–0·93) | *<0·001* | 0·96 (0·94–0·98) |
| *Model + baseline DM/HBP* | 1 | 0·95 (0·89–1·00) | 0·90 (0·85–0·96) | 0·82 (0·77–0·87) | *<0·001* | 0·95 (0·93–0·96) |
| ***Non-red meat UPF*** |  |  |  |  |  |  |
| *Model + alcohol* | 1 | 1·11 (1·04–1·18) | 1·15 (1·07–1·22) | 1·24 (1·16–1·32) | *<0·001* | 1·06 (1·04–1·08) |
| *Model + nutrients* | 1 | 1·08 (1·01–1·16) | 1·10 (1·03–1·18) | 1·16 (1·08–1·25) | *<0·001* | 1·04 (1·02–1·06) |
| *Model + baseline DM/HBP* | 1 | 1·11 (1·04–1·18) | 1·15 (1·07–1·22) | 1·24 (1·16–1·32) | *<0·001* | 1·06 (1·05–1·08) |
|  | **Coronary heart disease** | | | | | |
| ***Non-red meat non-UPF*** |  |  |  |  |  |  |
| *Model + alcohol* | 1 | 0·96 (0·9–1·03) | 0·91 (0·85–0·98) | 0·78 (0·72–0·84) | *<0·001* | 0·93 (0·92–0·95) |
| *Model + nutrients* | 1 | 0·98 (0·91–1·05) | 0·93 (0·86–1·00) | 0·81 (0·74–0·88) | *<0·001* | 0·94 (0·92–0·96) |
| *Model + baseline DM/HBP* | 1 | 0·96 (0·9–1·03) | 0·91 (0·85–0·97) | 0·77 (0·72–0·83) | *<0·001* | 0·93 (0·92–0·95 |
| ***Non-red meat UPF*** |  |  |  |  |  |  |
| *Model + alcohol* | 1 | 1·17 (1·08–1·26) | 1·23 (1·14–1·32) | 1·28 (1·19–1·39) | *<0·001* | 1·07 (1·05–1·09) |
| *Model + nutrients* | 1 | 1·15 (1·07–1·24) | 1·19 (1·10–1·29) | 1·22 (1·12–1·33) | *<0·001* | 1·05 (1·03–1·08) |
| *Model + baseline DM/HBP* | 1 | 1·18 (1·09–1·27) | 1·24 (1·15–1·33) | 1·30 (1·21–1·40) | *<0·001* | 1·07 (1·05–1·09) |
|  | **Cerebrovascular disease** | | | | | |
| ***Non-red meat non-UPF*** |  |  |  |  |  |  |
| *Model + alcohol* | 1 | 0·92 (0·81–1·04) | 0·89 (0·78–1·01) | 0·98 (0·87–1·11) | *0·663* | 0·99 (0·96–1·02) |
| *Model + nutrients* | 1 | 0·96 (0·85–1·08) | 0·96 (0·84–1·09) | 1·10 (0·96–1·26) | *0·213* | 1·03 (0·99–1·06) |
| *Model + baseline DM/HBP* | 1 | 0·93 (0·82–1·05) | 0·90 (0·80–1·02) | 1·01 (0·89–1·14) | 0·966 | 1·00 (0·97–1·03) |
| ***Non-red meat UPF*** |  |  |  |  |  |  |
| *Model + alcohol* | 1 | 0·94 (0·83–1·06) | 0·93 (0·82–1·05) | 1·09 (0·96–1·24) | *0·216* | 1·02 (0·98–1·05) |
| *Model + nutrients* | 1 | 0·90 (0·80–1·02) | 0·87 (0·76–0·99) | 0·98 (0·85–1·12) | *0·647* | 0·98 (0·95–1·02) |
| *Model + baseline DM/HBP* | 1 | 0·92 (0·82–1·04) | 0·90 (0·80–1·02) | 1·06 (0·94–1·20) | 0·393 | 1·01 (0·98–1·04) |
|  | **All cardiovascular diseases mortality** | | | | | |
| ***Non-red meat non-UPF*** |  |  |  |  |  |  |
| *Model + alcohol* | 1 | 0·96 (0·76–1·20) | 0·83 (0·65–1·06) | 0·70 (0·53–0·91) | *0·004* | 0·91 (0·85–0·98) |
| *Model + nutrients* | 1 | 0·96 (0·76–1·21) | 0·83 (0·65–1·07) | 0·71 (0·53–0·94) | *0·012* | 0·92 (0·85–0·99) |
| *Model + baseline DM/HBP* | 1 | 0·97 (0·78–1·22) | 0·85 (0·67–1·08) | 0·72 (0·56–0·93) | 0·007 | 0·92 (0·86–0·98) |
| ***Non-red meat UPF*** |  |  |  |  |  |  |
| *Model + alcohol* | 1 | 1·13 (0·88–1·46) | 1·17 (0·9–1·51) | 1·36 (1·05–1·76) | *0·023* | 1·08 (1·01–1·16) |
| *Model + nutrients* | 1 | 1·12 (0·87–1·46) | 1·15 (0·89–1·50) | 1·34 (1·01–1·77) | *0·049* | 1·08 (1·00–1·16) |
| *Model + baseline DM/HBP* | 1 | 1·14 (0·88–1·47) | 1·18 (0·91–1·51) | 1·36 (1·06–1·74) | 0·018 | 1·08 (1·01–1·15) |
|  | **Coronary heart disease mortality** | | | | | |
| ***Non-red meat non-UPF*** |  |  |  |  |  |  |
| *Model + alcohol* | 1 | 0·95 (0·72–1·26) | 0·87 (0·65–1·16) | 0·57 (0·40–0·80) | *0·002* | 0·86 (0·79–0·93) |
| *Model + nutrients* | 1 | 0·98 (0·74–1·30) | 0·92 (0·68–1·24) | 0·63 (0·43–0·91) | *0·022* | 0·89 (0·80–0·97) |
| *Model + baseline DM/HBP* | 1 | 0·98 (0·75–1·30) | 0·91 (0·68–1·21) | 0·60 (0·43–0·84) | 0·004 | 0·88 (0·81–0·95) |
| ***Non-red meat UPF*** |  |  |  |  |  |  |
| *Model + alcohol* | 1 | 1·35 (0·97–1·88) | 1·56 (1·13–2·16) | 1·56 (1·12–2·18) | *0·008* | 1·13 (1·04–1·23) |
| *Model + nutrients* | 1 | 1·30 (0·93–1·82) | 1·46 (1·05–2·04) | 1·42 (0·99–2·03) | *0·053* | 1·11 (1·01–1·21) |
| *Model + baseline DM/HBP* | 1 | 1·36 (0·98–1·89) | 1·56 (1·13–2·15) | 1·53 (1·11–2·12) | 0·008 | 1·12 (1·04–1·22) |
|  | **Cerebrovascular disease mortality** | | | | | |
| ***Non-red meat non-UPF*** |  |  |  |  |  |  |
| *Model + alcohol* | 1 | 0·96 (0·64–1·43) | 0·75 (0·48–1·16) | 0·95 (0·62–1·45) | 0·557 | 1·01 (0·90–1·13) |
| *Model + nutrients* | 1 | 0·91 (0·61–1·37) | 0·68 (0·43–1·06) | 0·80 (0·50–1·28) | 0·206 | 0·97 (0·86–1·10) |
| *Model + baseline DM/HBP* | 1 | 0·95 (0·64–1·42) | 0·74 (0·48–1·14) | 0·93 (0·62–1·41) | *0·504* | 1·00 (0·90–1·12) |
| ***Non-red meat UPF*** |  |  |  |  |  |  |
| *Model + alcohol* | 1 | 0·86 (0·57–1·3) | 0·67 (0·43–1·05) | 1·11 (0·74–1·68) | 0·816 | 0·99 (0·88–1·11) |
| *Model + nutrients* | 1 | 0·91 (0·60–1·39) | 0·74 (0·47–1·18) | 1·30 (0·82–2·06) | 0·408 | 1·03 (0·90–1·17) |
| *Model + baseline DM/HBP* | 1 | 0·87 (0·57–1·31) | 0·68 (0·44–1·06) | 1·15 (0·77–1·70) | 0·701 | 1·00 (0·90–1·12) |
| Q = Quartile. UPF = Ultra-processed foods. DM = Type 2 diabetes mellitus. HBP = High blood pressure. | | | | | | |
| Cox proportional hazards models with age as the underlying timescale. Adjusted by sex, ethnic (white, non-white), family history of CVD (no, mother or father, mother and father), BMI (continuous), physical activity (low, moderate, high, missing), smoking status (never, previous, current), index of multiple deprivation (quintile), and region (London, South East, South West, East Midlands, West Midlands, Yorkshire & the Humber, North East, North West, Wales, Scotland). Analysis for risk of CVD were stratified by sex, family history of CVD and smoking status. Analysis for the CVD death were stratified by sex and ethnic. Model was additionally adjusted for alcohol intake (g/day), nutrients (% free sugar of total energy, % saturated fat of total energy, sodium density and fibre density) and status of type II diabetes mellitus or high blood pressure at baseline. | | | | | | |

| **Table S8. Association** **between the dietary contribution of foods groups that take into account both the plant or animal origin of foods and food processing categories, and fatal and non-fatal cardiovascular events from in the UK Biobank cohort, considering the dietary contribution of total grams (n = 118,397)** | | | | | | |
| --- | --- | --- | --- | --- | --- | --- |
|  | **Dietary contribution (% of total grams)** | | | | | |
| **Food groups** | **Q1** | **Q2** | **Q3** | **Q4** |  | **Continuous (10% increase in the contribution)** |
|  | *HR (95% CI)* | | | | *p for trend* | *HR (95% CI)* |
|  | **All cardiovascular diseases** | | | | | |
| ***Plant-sourced non-UPF*** | 1 | 0·90 (0·85–0·96) | 0·92 (0·86–0·97) | 0·87 (0·81–0·93) | *<0·001* | 0·95 (0·94–0·97) |
| ***Plant-sourced UPF*** | 1 | 1·10 (1·04–1·18) | 1·13 (1·06–1·21) | 1·21 (1·14–1·30) | *<0·001* | 1·09 (1·07–1·11) |
| ***All plant-sourced foods*** | 1 | 1·04 (0·98–1·11) | 1·03 (0·97–1·10) | 1·02 (0·95–1·08) | *0·716* | 1·01 (0·98–1·04) |
| ***All UPF*** | 1 | 1·15 (1·08–1·23) | 1·15 (1·07–1·22) | 1·25 (1·17–1·34) | *<0·001* | 1·08 (1·06–1·10) |
|  | **Coronary heart disease** | | | | | |
| ***Plant-sourced non-UPF*** | 1 | 0·89 (0·83–0·95) | 0·90 (0·84–0·97) | 0·83 (0·77–0·90) | *<0·001* | 0·94 (0·93–0·96) |
| ***Plant-sourced UPF*** | 1 | 1·16 (1·07–1·25) | 1·22 (1·13–1·31) | 1·27 (1·17–1·37) | *<0·001* | 1·10 (1·08–1·13) |
| ***All plant-sourced foods*** | 1 | 1·05 (0·98–1·13) | 1·04 (0·97–1·12) | 0·99 (0·92–1·07) | *0·805* | 1·00 (0·97–1·04) |
| ***All UPF*** | 1 | 1·23 (1·14–1·33) | 1·20 (1·11–1·30) | 1·34 (1·24–1·44) | *<0·001* | 1·10 (1·07–1·12) |
|  | **Cerebrovascular disease** | | | | | |
| ***Plant-sourced non-UPF*** | 1 | 0·95 (0·84–1·07) | 0·95 (0·84–1·07) | 0·96 (0·85–1·09) | *0·555* | 0·98 (0·95–1·01) |
| ***Plant-sourced UPF*** | 1 | 0·95 (0·84–1·07) | 0·95 (0·84–1·07) | 1·09 (0·97–1·24) | *0·203* | 1·05 (1·00–1·09) |
| ***All plant-sourced foods*** | 1 | 1·02 (0·91–1·15) | 0·99 (0·88–1·12) | 1·08 (0·96–1·22) | *0·300* | 1·02 (0·97–1·08) |
| ***All UPF*** | 1 | 0·94 (0·84–1·07) | 1·01 (0·90–1·15) | 1·04 (0·92–1·19) | *0·328* | 1·04 (1·00–1·08) |
|  | **All cardiovascular diseases mortality** | | | | | |
| ***Plant-sourced non-UPF*** | 1 | 0·76 (0·6–0·96) | 0·74 (0·58–0·93) | 0·61 (0·48–0·79) | *<0·001* | 0·86 (0·81–0·91) |
| ***Plant-sourced UPF*** | 1 | 0·94 (0·72–1·23) | 1·27 (0·99–1·63) | 1·45 (1·12–1·86) | *0·001* | 1·22 (1·13–1·32) |
| ***All plant-sourced foods*** | 1 | 0·95 (0·75–1·20) | 0·84 (0·66–1·07) | 0·81 (0·63–1·03) | *0·055* | 0·92 (0·83–1·01) |
| ***All UPF*** | 1 | 1·19 (0·92–1·54) | 1·28 (0·99–1·66) | 1·55 (1·19–2·01) | *<0·001* | 1·19 (1·11–1·28) |
|  | **Coronary heart disease mortality** | | | | | |
| ***Plant-sourced non-UPF*** | 1 | 0·74 (0·56–0·98) | 0·66 (0·49–0·88) | 0·55 (0·40–0·75) | *<0·001* | 0·83 (0·78–0·90) |
| ***Plant-sourced UPF*** | 1 | 1·32 (0·94–1·87) | 1·66 (1·19–2·32) | 1·93 (1·38–2·70) | *<0·001* | 1·27 (1·17–1·39) |
| ***All plant-sourced foods*** | 1 | 1·05 (0·79–1·39) | 0·85 (0·63–1·14) | 0·79 (0·58–1·07) | *0·061* | 0·92 (0·81–1·04) |
| ***All UPF*** | 1 | 1·49 (1·06–2·09) | 1·66 (1·18–2·32) | 1·99 (1·42–2·79) | *0·004* | 1·23 (1·13–1·33) |
|  | **Cerebrovascular disease mortality** | | | | | |
| ***Plant-sourced non-UPF*** | 1 | 0·81 (0·53–1·23) | 0·92 (0·61–1·38) | 0·76 (0·49–1·18) | *0·334* | 0·91 (0·82–1·01) |
| ***Plant-sourced UPF*** | 1 | 0·57 (0·37–0·88) | 0·90 (0·61–1·34) | 0·96 (0·63–1·45) | *0·810* | 1·10 (0·96–1·27) |
| ***All plant-sourced foods*** | 1 | 0·77 (0·52–1·16) | 0·82 (0·55–1·23) | 0·84 (0·56–1·27) | *0·463* | 0·91 (0·76–1·08) |
| ***All UPF*** | 1 | 0·87 (0·58–1·31) | 0·87 (0·57–1·33) | 1·05 (0·68–1·61) | *0·881* | 1·12 (0·98–1·27) |
| Q = Quartile. UPF = Ultra-processed foods. | | | | | | |
| Cox proportional hazards models with age as the underlying timescale. Adjusted by sex, ethnic (white, non-white), family history of CVD (no, mother or father, mother and father), BMI (continuous), physical activity (low, moderate, high, missing), smoking status (never, previous, current), index of multiple deprivation (quintile), region (London, South East, South West, East Midlands, West Midlands, Yorkshire & the Humber, North East, North West, Wales, Scotland), and total energy intake (kcal/day). Analysis for risk of CVD were stratified by sex, family history of CVD and smoking status. Analysis for the CVD death were stratified by sex and ethnic. | | | | | | |

| **Table S9. Association** **between the dietary contribution of food groups that take into account both the red meat or non-red meat origin of foods and food processing categories and fatal and non-fatal cardiovascular events from in the UK Biobank cohort, considering the dietary contribution of total grams (n = 118,397).** | | | | | | |
| --- | --- | --- | --- | --- | --- | --- |
|  | **Dietary contribution (% of total grams)** | | | | | |
| **Food groups** | **Q1** | **Q2** | **Q3** | **Q4** |  | **Continuous (10% increase in the contribution)** |
|  | *HR (95% CI)* | | | | *p for trend* | *HR (95% CI)* |
|  | **All cardiovascular diseases** | | | | | |
| ***Non-red meat non-UPF*** | 1 | 0·90 (0·84–0·95) | 0·90 (0·85–0·96) | 0·78 (0·73–0·83) | *<0·001* | 0·92 (0·91–0·94) |
| ***Non-red meat UPF*** | 1 | 1·14 (1·07–1·21) | 1·14 (1·06–1·21) | 1·28 (1·20–1·37) | *<0·001* | 1·09 (1·06–1·11) |
| ***All non-red meat*** | 1 | 0·99 (0·93–1·05) | 0·99 (0·94–1·06) | 0·96 (0·90–1·02) | *0·247* | 0·97 (0·88–1·06) |
|  | **Coronary heart disease** | | | | | |
| ***Non-red meat non-UPF*** | 1 | 0·90 (0·84–0·96) | 0·90 (0·84–0·97) | 0·73 (0·68–0·79) | *<0·001* | 0·91 (0·84–0·93) |
| ***Non-red meat UPF*** | 1 | 1·21 (1·12–1·30) | 1·19 (1·10–1·28) | 1·36 (1·26–1·47) | *<0·001* | 1·10 (1·08–1·12) |
| ***All non-red meat*** | 1 | 0·99 (0·92–1·06) | 0·99 (0·93–1·07) | 0·95 (0·89–1·03) | *0·269* | 0·96 (0·87–1·07) |
|  | **Cerebrovascular disease** | | | | | |
| ***Non-red meat non-UPF*** | 1 | 0·90 (0·80–1·02) | 0·88 (0·78–1·00) | 0·93 (0·82–1·06) | *0·271* | 0·97 (0·93–1·00) |
| ***Non-red meat UPF*** | 1 | 0·96 (0·85–1·08) | 1·01 (0·89–1·14) | 1·07 (0·94–1·21) | *0·224* | 1·04 (1·00–1·08) |
| ***All non-red meat*** | 1 | 1·02 (0·90–1·14) | 0·99 (0·87–1·11) | 1·01 (0·89–1·15) | *1·000* | 1·03 (0·86–1·24) |
|  | **All cardiovascular diseases mortality** | | | | | |
| ***Non-red meat non-UPF*** | 1 | 0·82 (0·65–1·03) | 0·64 (0·50–0·82) | 0·63 (0·49–0·82) | *0·002* | 0·84 (0·78–0·89) |
| ***Non-red meat UPF*** | 1 | 1·11 (0·86–1·44) | 1·25 (0·97–1·62) | 1·51 (1·17–1·95) | *0·001* | 1·19 (1·11–1·28) |
| ***All non-red meat*** | 1 | 0·75 (0·60–0·95) | 0·72 (0·57–0·92) | 0·85 (0·67–1·09) | *0·106* | 0·68 (0·48–0·94) |
|  | **Coronary heart disease mortality** | | | | | |
| ***Non-red meat non-UPF*** | 1 | 0·79 (0·60–1·04) | 0·65 (0·48–0·87) | 0·53 (0·38–0·73) | *<0·001* | 0·81 (0·75–0·88) |
| ***Non-red meat UPF*** | 1 | 1·46 (1·04–2·05) | 1·67 (1·19–2·33) | 1·98 (1·42–2·77) | *<0·001* | 1·23 (1·13–1·33) |
| ***All non-red meat*** | 1 | 0·77 (0·58–1·02) | 0·69 (0·51–0·92) | 0·81 (0·60–1·10) | *0·072* | 0·63 (0·42–0·94) |
|  | **Cerebrovascular disease mortality** | | | | | |
| ***Non-red meat non-UPF*** | 1 | 0·88 (0·59–1·32) | 0·63 (0·40–0·98) | 0·85 (0·55–1·29) | *0·245* | 0·89 (0·79–1·01) |
| ***Non-red meat UPF*** | 1 | 0·75 (0·49–1·13) | 0·82 (0·54–1·24) | 1·01 (0·66–1·54) | *0·934* | 1·12 (0·98–1·27) |
| ***All non-red meat*** | 1 | 0·72 (0·48–1·09) | 0·80 (0·53–1·19) | 0·94 (0·62–1·40) | *0·798* | 0·79 (0·43–1·45) |
| Q = Quartile. UPF = Ultra-processed foods. | | | | | | |
| Cox proportional hazards models with age as the underlying timescale. Adjusted by sex, ethnic (white, non-white), family history of CVD (no, mother or father, mother and father), BMI (continuous), physical activity (low, moderate, high, missing), smoking status (never, previous, current), index of multiple deprivation (quintile), region (London, South East, South West, East Midlands, West Midlands, Yorkshire & the Humber, North East, North West, Wales, Scotland), and total energy intake (kcal/day). Analysis for risk of CVD were stratified by sex, family history of CVD and smoking status. Analysis for the CVD death were stratified by sex and ethnic. | | | | | | |

| **Table S10.** **Association between the dietary contribution of foods groups that take into account both the plant or animal origin of foods and food processing categories, and fatal and non-fatal cardiovascular events from in the UK Biobank cohort, excluded participants with follow-up time <2 years (n=117,086).** | | | | | | |
| --- | --- | --- | --- | --- | --- | --- |
|  | **Dietary contribution (% of total energy)** | | | | | |
| **Food groups** | **Q1** | **Q2** | **Q3** | **Q4** |  | **Continuous (10% increase in the contribution)** |
|  | *HR (95% CI)* | | | | *p for trend* | *HR (95% CI)* |
|  | **All cardiovascular diseases** | | | | | |
|  | *n for cases/non-cases = 6,495/110,591* | | | | | |
| ***Plant-sourced non-UPF*** | 1 | 0·90 (0·84–0·96) | 0·87 (0·81–0·93) | 0·82 (0·77–0·88) | *<0·001* | 0·94 (0·92–0·96) |
| ***Plant-sourced UPF*** | 1 | 1·07 (1·00–1·15) | 1·15 (1·07–1·23) | 1·18 (1·10–1·27) | *<0·001* | 1·06 (1·04–1·08) |
| ***All plant-sourced foods*** | 1 | 1·03 (0·96–1·11) | 0·97 (0·91–1·04) | 1·02 (0·95–1·09) | *0·933* | 1·01 (0·99–1·04) |
| ***All UPF*** | 1 | 1·07 (0·99–1·15) | 1·14 (1·06–1·22) | 1·22 (1·14–1·31) | *<0·001* | 1·06 (1·04–1·08) |
|  | **Coronary heart disease** | | | | | |
|  | *n for cases/non-cases =4,974/112,391* | | | | | |
| ***Plant-sourced non-UPF*** | 1 | 0·89 (0·82–0·96) | 0·85 (0·79–0·92) | 0·78 (0·72–0·85) | *<0·001* | 0·92 (0·90–0·95) |
| ***Plant-sourced UPF*** | 1 | 1·11 (1·02–1·20) | 1·21 (1·11–1·31) | 1·22 (1·13–1·33) | *<0·001* | 1·07 (1·04–1·09) |
| ***All plant-sourced foods*** | 1 | 1·06 (0·98–1·15) | 0·97 (0·89–1·05) | 1·01 (0·93–1·10) | *0·639* | 1·00 (0·96–1·03) |
| ***All UPF*** | 1 | 1·14 (1·05–1·23) | 1·19 (1·10–1·30) | 1·30 (1·20–1·41) | *<0·001* | 1·07 (1·05–1·09) |
|  | **Cerebrovascular disease** | | | | | |
|  | n for cases/non-cases = 1,813/116,285 | | | | | |
| ***Plant-sourced non-UPF*** | 1 | 0·95 (0·83–1·08) | 0·97 (0·85–1·10) | 0·96 (0·84–1·09) | *0·601* | 1·00 (0·96–1·04) |
| ***Plant-sourced UPF*** | 1 | 0·99 (0·87–1·12) | 0·97 (0·85–1·11) | 1·08 (0·95–1·24) | *0·291* | 1·02 (0·98–1·06) |
| ***All plant-sourced foods*** | 1 | 0·95 (0·83–1·08) | 1·01 (0·88–1·14) | 1·02 (0·89–1·17) | *0·579* | 1·03 (0·99–1·08) |
| ***All UPF*** | 1 | 0·92 (0·80–1·05) | 0·97 (0·85–1·11) | 1·01 (0·88–1·15) | *0·703* | 1·01 (0·97–1·04) |
|  | **All cardiovascular diseases mortality** | | | | | |
|  | *n for cases /non-cases = 480/116,606* | | | | | |
| ***Plant-sourced non-UPF*** | 1 | 0·89 (0·71–1·13) | 0·79 (0·61–1·01) | 0·59 (0·45–0·77) | *<0·001* | 0·86 (0·79–0·93) |
| ***Plant-sourced UPF*** | 1 | 1·03 (0·78–1·37) | 1·44 (1·10–1·88) | 1·56 (1·20–2·03) | *<0·001* | 1·14 (1·06–1·22) |
| ***All plant-sourced foods*** | 1 | 1·13 (0·88–1·44) | 0·91 (0·70–1·17) | 0·91 (0·70–1·19) | *0·242* | 0·92 (0·83–1·03) |
| ***All UPF*** | 1 | 1·33 (1·02–1·74) | 1·30 (0·99–1·71) | 1·46 (1·12–1·91) | *0·012* | 1·09 (1·02–1·17) |
|  | **Coronary heart disease mortality** | | | | | |
|  | *n for cases/non-cases = 315/116,771* | | | | | |
| ***Plant-sourced non-UPF*** | 1 | 0·80 (0·60–1·07) | 0·72 (0·54–0·97) | 0·44 (0·31–0·63) | *<0·001* | 0·79 (0·71–0·87) |
| ***Plant-sourced UPF*** | 1 | 1·41 (0·97–2·05) | 1·93 (1·35–2·75) | 2·04 (1·43–2·91) | *<0·001* | 1·21 (1·11–1·32) |
| ***All plant-sourced foods*** | 1 | 1·17 (0·86–1·58) | 0·84 (0·60–1·16) | 0·89 (0·64–1·23) | *0·177* | 0·92 (0·81–1·04) |
| ***All UPF*** | 1 | 1·55 (1·09–2·20) | 1·55 (1·10–2·20) | 1·73 (1·23–2·44) | *0·004* | 1·14 (1·05–1·24) |
|  | **Cerebrovascular disease mortality** | | | | | |
|  | *n for cases/non-cases = 165/116,921* | | | | | |
| ***Plant-sourced non-UPF*** | 1 | 1·13 (0·74–1·72) | 0·96 (0·62–1·49) | 0·97 (0·62–1·52) | *0·715* | 1·02 (0·89–1·16) |
| ***Plant-sourced UPF*** | 1 | 0·66 (0·42–1·05) | 0·96 (0·63–1·46) | 1·09 (0·71–1·66) | *0·427* | 1·01 (0·89–1·14) |
| ***All plant-sourced foods*** | 1 | 1·06 (0·70–1·60) | 1·05 (0·69–1·60) | 0·96 (0·61–1·52) | *0·899* | 1·04 (0·89–1·21) |
| ***All UPF*** | 1 | 1·06 (0·69–1·63) | 0·99 (0·63–1·54) | 1·12 (0·72–1·74) | *0·706* | 1·00 (0·90–1·13) |
| Q = Quartile. UPF = Ultra-processed foods. | | | | | | |
| Mean follow-up times were 9·2 for overall cardiovascular disease (1,074,782 person-years), 9·2 coronary heart disease (1,082,457 person-years), and 9·3 for cerebrovascular diseases (1,101,407 person-years). Mean follow-up times were 9·3 for mortality for cardiovascular disease (1,091,051 person-years), coronary heart disease (1,091,051 person-years), and cerebrovascular diseases (1,091,051 person-years). | | | | | | |
| Cox proportional hazards models with age as the underlying timescale. Adjusted by sex, ethnic (white, non-white), family history of CVD (no, mother or father, mother and father), BMI (continuous), physical activity (low, moderate, high, missing), smoking status (never, previous, current), index of multiple deprivation (quintile), and region (London, South East, South West, East Midlands, West Midlands, Yorkshire & the Humber, North East, North West, Wales, Scotland). Analysis for risk of CVD were stratified by sex, family history of CVD and smoking status. Analysis for the CVD death were stratified by sex and ethnic. | | | | | | |

| **Table S11. Association** **between the dietary contribution of food groups that take into account both the red meat or non-red meat origin of foods and food processing categories and fatal and non-fatal cardiovascular events from in the UK Biobank cohort, excluded participants with follow-up time <2 years (n=117,086).** | | | | | | |
| --- | --- | --- | --- | --- | --- | --- |
|  | **Dietary contribution (% of total energy)** | | | | | |
| **Food groups** | **Q1** | **Q2** | **Q3** | **Q4** |  | **Continuous (10% increase in the contribution)** |
|  | *HR (95% CI)* | | | | *p for trend* | *HR (95% CI)* |
|  | **All cardiovascular diseases** | | | | | |
| ***Non-red meat non-UPF*** | 1 | 0·94 (0·88–1·00) | 0·88 (0·82–0·94) | 0·82 (0·76–0·88) | *<0·001* | 0·94 (0·93–0·96) |
| ***Non-red meat UPF*** | 1 | 1·10 (1·02–1·18) | 1·12 (1·04–1·20) | 1·25 (1·17–1·34) | *<0·001* | 1·06 (1·04–1·08) |
| ***All non-red meat*** | 1 | 1·02 (0·95–1·09) | 1·00 (0·94–1·07) | 0·96 (0·90–1·04) | 0·306 | 0·99 (0·95–1·04) |
|  | **Coronary heart disease** | | | | | |
| ***Non-red meat non-UPF*** | 1 | 0·95 (0·88–1·02) | 0·87 (0·81–0·94) | 0·77 (0·71–0·84) | *<0·001* | 0·93 (0·91–0·95) |
| ***Non-red meat UPF*** | 1 | 1·16 (1·07–1·26) | 1·21 (1·12–1·31) | 1·31 (1·21–1·42) | *<0·001* | 1·07 (1·05–1·09) |
| ***All non-red meat*** | 1 | 1·02 (0·94–1·10) | 0·97 (0·90–1·05) | 0·95 (0·87–1·03) | *0·138* | 0·98 (0·93–1·03) |
|  | **Cerebrovascular disease** | | | | | |
| ***Non-red meat non-UPF*** | 1 | 0·93 (0·81–1·06) | 0·92 (0·80–1·05) | 1·00 (0·88–1·14) | 0·998 | 1·00 (0·96–1·03) |
| ***Non-red meat UPF*** | 1 | 0·95 (0·83–1·08) | 0·90 (0·79–1·03) | 1·09 (0·96–1·24) | 0·294 | 1·01 (0·98–1·05) |
| ***All non-red meat*** | 1 | 1·03 (0·91–1·18) | 1·08 (0·95–1·23) | 1·03 (0·90–1·18) | 0·506 | 1·06 (0·97–1·15) |
|  | **All cardiovascular diseases mortality** | | | | | |
| ***Non-red meat non-UPF*** | 1 | 0·93 (0·74–1·18) | 0·79 (0·61–1·01) | 0·65 (0·50–0·85) | 0·001 | 0·90 (0·84–0·96) |
| ***Non-red meat UPF*** | 1 | 1·18 (0·9–1·54) | 1·17 (0·89–1·52) | 1·44 (1·11–1·86) | 0·010 | 1·09 (1·02–1·17) |
| ***All non-red meat*** | 1 | 0·80 (0·63–1·03) | 0·80 (0·62–1·02) | 0·88 (0·68–1·14) | 0·259 | 0·90 (0·77–1·04) |
|  | **Coronary heart disease mortality** | | | | | |
| ***Non-red meat non-UPF*** | 1 | 0·94 (0·70–1·25) | 0·84 (0·62–1·14) | 0·53 (0·37–0·76) | *0·001* | 0·85 (0·79–0·93) |
| ***Non-red meat UPF*** | 1 | 1·42 (1·00–2·01) | 1·52 (1·08–2·13) | 1·65 (1·18–2·32) | *0·005* | 1·14 (1·05–1·24) |
| ***All non-red meat*** | 1 | 0·87 (0·65–1·16) | 0·72 (0·53–0·99) | 0·86 (0·62–1·18) | *0·158* | 0·88 (0·73–1·06) |
|  | **Cerebrovascular disease mortality** | | | | | |
| ***Non-red meat non-UPF*** | 1 | 0·92 (0·61–1·39) | 0·69 (0·44–1·07) | 0·86 (0·56–1·32) | 0·289 | 0·98 (0·88–1·10) |
| ***Non-red meat UPF*** | 1 | 0·88 (0·57–1·36) | 0·73 (0·46–1·15) | 1·19 (0·78–1·79) | 0·580 | 1·01 (0·90–1·13) |
| ***All non-red meat*** | 1 | 0·68 (0·44–1·06) | 0·94 (0·62–1·41) | 0·93 (0·60–1·42) | 0·970 | 0·94 (0·71–1·22) |
| Q = Quartile. UPF = Ultra-processed foods | | | | | | |
| Cox proportional hazards models with age as the underlying timescale. Adjusted by sex, ethnic (white, non-white), family history of CVD (no, mother or father, mother and father), BMI (continuous), physical activity (low, moderate, high, missing), smoking status (never, previous, current), index of multiple deprivation (quintile), and region (London, South East, South West, East Midlands, West Midlands, Yorkshire & the Humber, North East, North West, Wales, Scotland). Analysis for risk of CVD were stratified by sex, family history of CVD and smoking status. Analysis for the CVD death were stratified by sex and ethnic. | | | | | | |


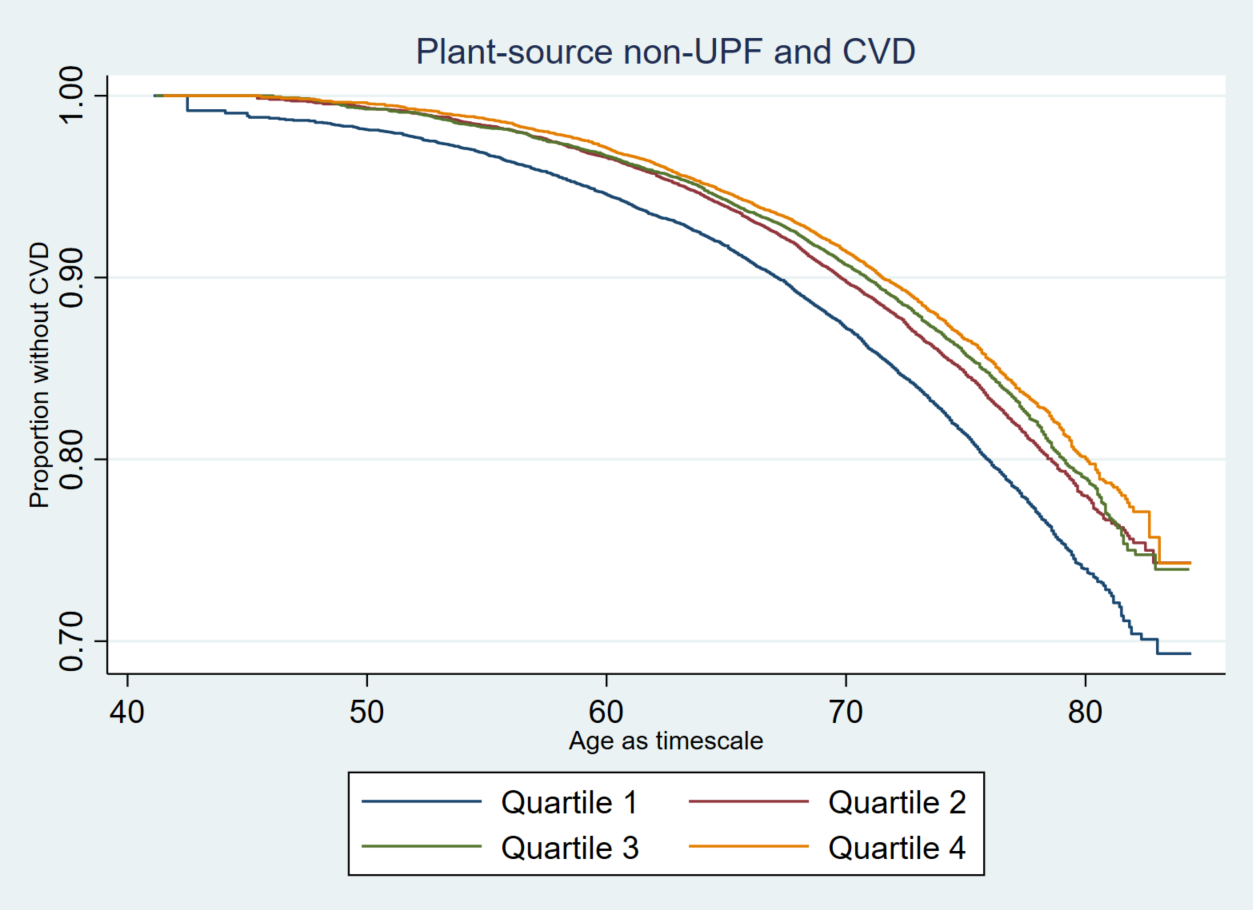

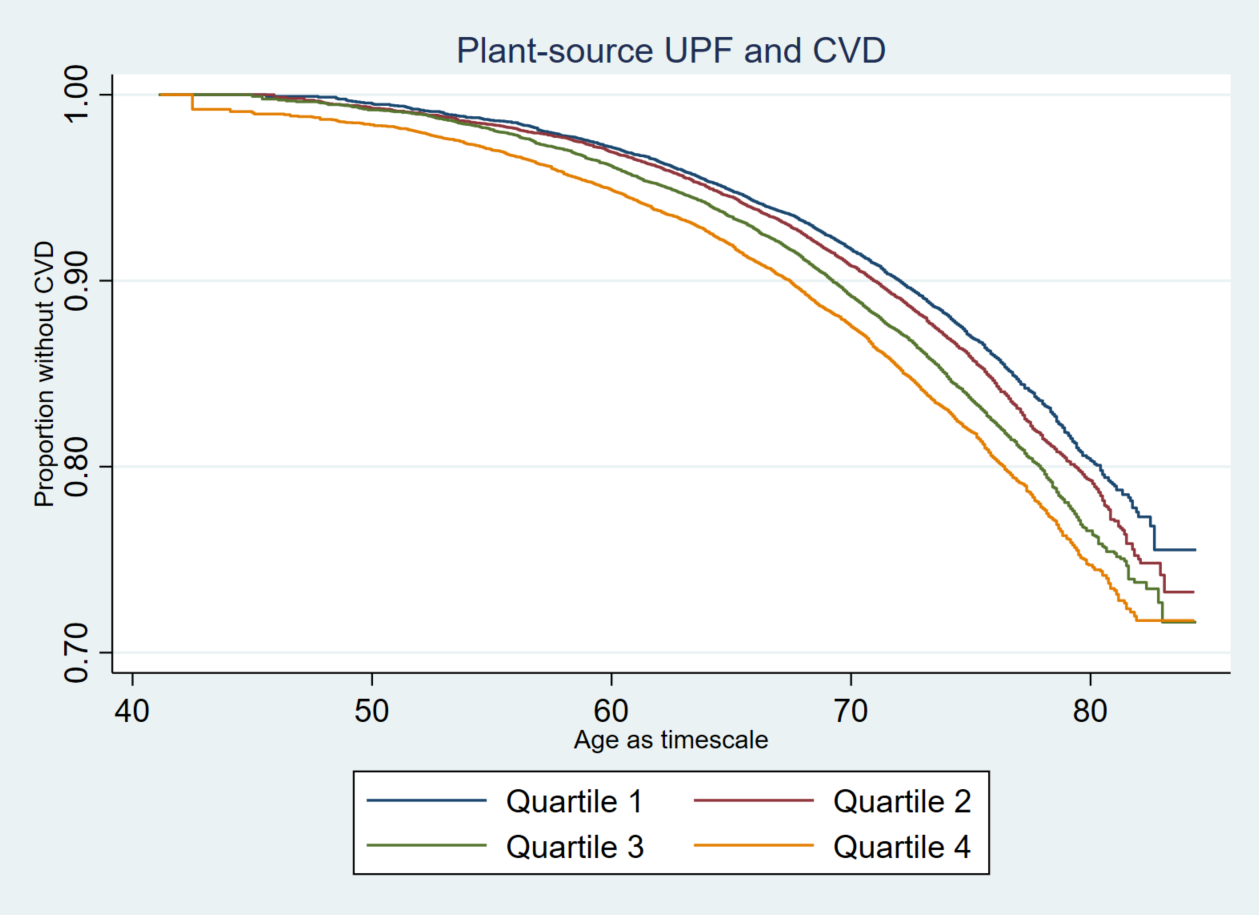


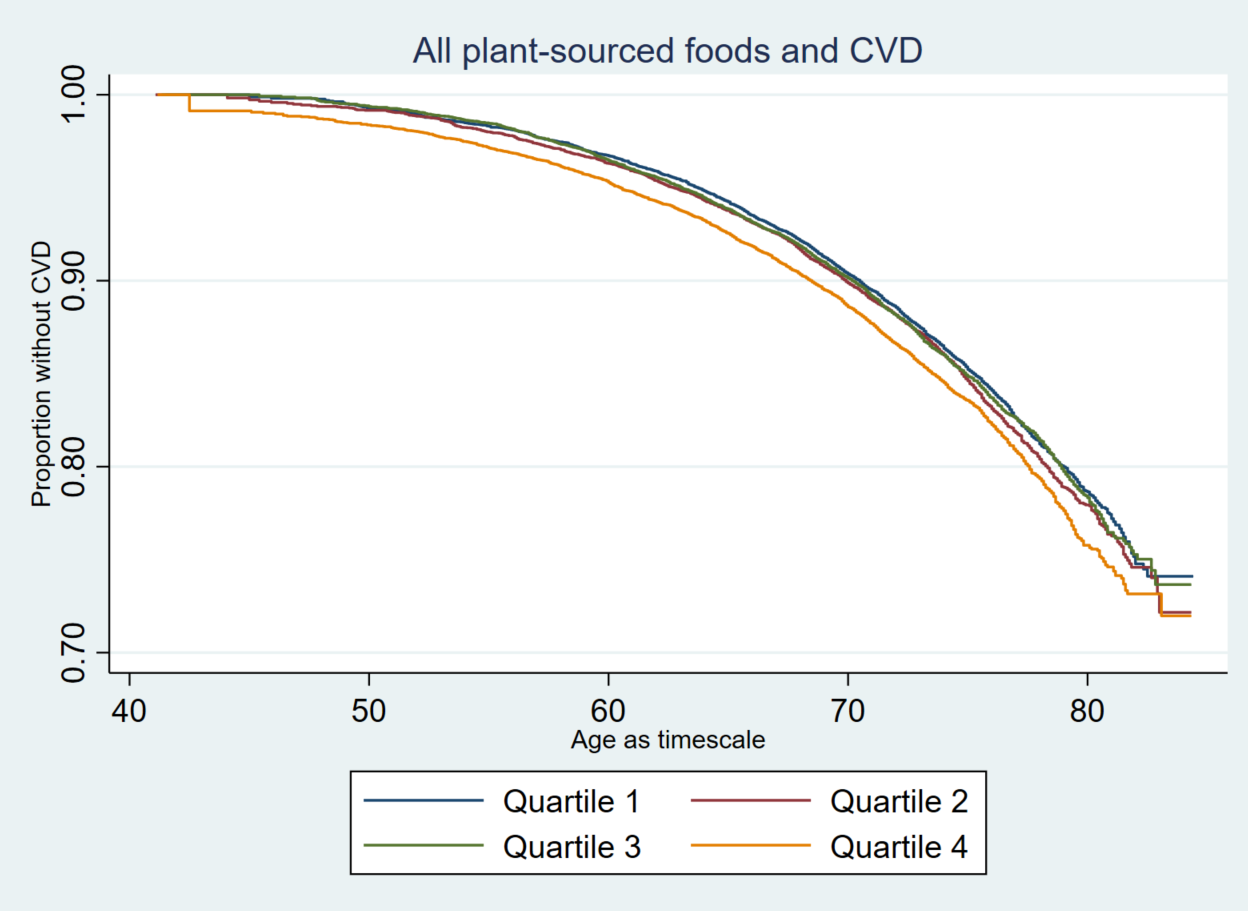

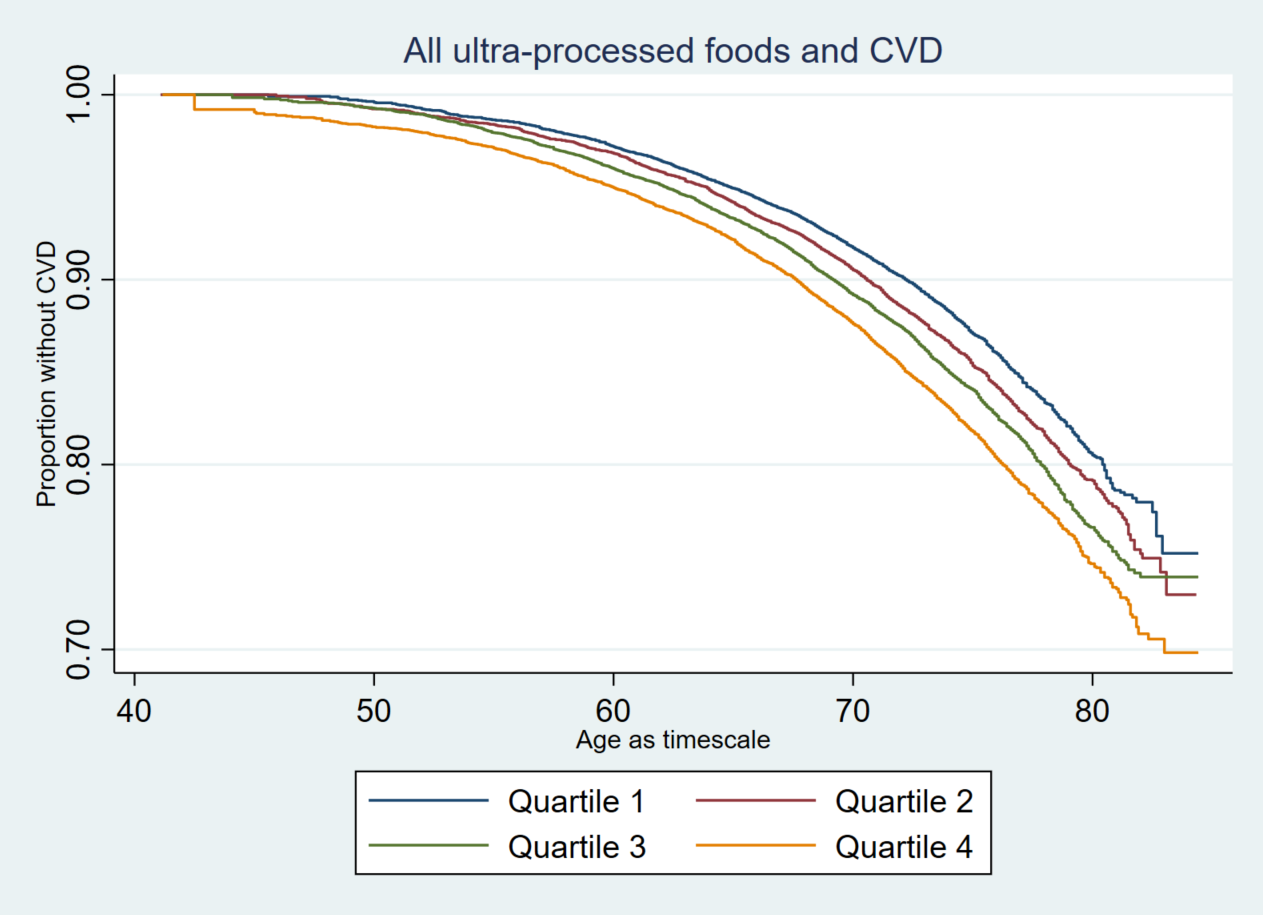


Figure S2. Kaplan-Meier plots
